# Supplementary material for: Health itinerary-related survival of children under-five with severe malaria or bloodstream infection, DR Congo
Source: PLoS Negl Trop Dis. 2023 Mar 6;17(3):e0011156. doi: 10.1371/journal.pntd.0011156 (PMC10019685; doi:10.1371/journal.pntd.0011156)
Supplement: S1 Table — (DOCX) [file pntd.0011156.s001.docx]

Supplementary Materials: Health itinerary-related survival of children under-five with severe malaria or bloodstream infection, DR Congo

Bieke Tack ^1,2,3^, Daniel Vita ^4^, José Nketo ^5^, Naomie Wasolua ^4^, Nathalie Ndengila ^4^, Natacha Herssens ^1^, Emmanuel Ntangu ^4^, Grace Kasidiko ^4^, Gaëlle Nkoji-Tunda ^6,7^, Marie-France Phoba ^6,7^, Justin Im ^8^, Hyon Jin Jeon ^8,9^, Florian Marks ^8-12^, Jaan Toelen ^3,9^, Octavie Lunguya ^6,7^ and Jan Jacobs ^1,2^

**Affiliations:**

1. Department of Clinical Sciences, Institute of Tropical Medicine, Antwerp, Belgium
2. Department of Microbiology, Immunology and Transplantation, KU Leuven, Leuven, Belgium
3. Department of Pediatrics, University Hospitals UZ Leuven, Leuven, Belgium
4. Hôpital Général de Référence Saint Luc de Kisantu, Kisantu, Democratic Republic of the Congo
5. Zone de Santé Kisantu, Kisantu, Democratic Republic of the Congo
6. Department of Microbiology, Institut National de Recherche Biomédicale, Kinshasa, Democratic Republic of the Congo
7. Department of Medical Biology, University Teaching Hospital of Kinshasa, Kinshasa, Democratic Republic of the Congo
8. International Vaccine Institute, Seoul, Republic of Korea
9. Cambridge Institute of Therapeutic Immunology and Infectious Disease, University of Cambridge School of Clinical Medicine, Cambridge, UK
10. Heidelberg Institute of Global Health, University of Heidelberg, Heidelberg, Germany
11. Madagascar Institute for Vaccine Research, University of Antananarivo, Antananarivo, Madagascar
12. Department of Development and Regeneration, KU Leuven, 3000 Leuven, Belgium

**Corresponding author:**

Bieke Tack, [btack@itg.be](mailto:btack@itg.be)

**Supplementary Table S1.** Indications for blood culture sampling: if children fulfil both criteria when they arrive at the hospital, a blood culture is routinely sampled upon admission of the child.

| **CRITERIUM 1: fever or hypothermia** | | | |
| --- | --- | --- | --- |
|  | Rectal | Axillary | Tympanic |
| Fever upon presentation | > 38.0 °C | > 37.5°C | > 37.5°C |
| Hypothermia upon presentation | < 36.0 °C | ≤ 35.5°C | ≤ 35.5°C |
| Subjective fever in the past 48 hours (as reported by the caretaker) | | | |
| **CRITERIUM 2: Minimum 1 severity sign present** | | | |
| Quick SOFA score ≥ 1 | Hypotension | | |
|  | Confusion | | |
|  | Increased respiratory rate | | |
| Suspicion of severe localized infection | Pneumonia | | |
|  | Meningitis | | |
|  | Osteomyelitis | | |
|  | Complicated urinary tract infection | | |
|  | Abscess | | |
|  | Severe skin or soft tissue infection | | |
|  | Abdominal infection | | |
| Suspicion of other severe systemic infection | Severe malaria | | |
|  | Typhoid fever | | |
