# Supplementary material for: Health itinerary-related survival of children under-five with severe malaria or bloodstream infection, DR Congo
Source: PLoS Negl Trop Dis. 2023 Mar 6;17(3):e0011156. doi: 10.1371/journal.pntd.0011156 (PMC10019685; doi:10.1371/journal.pntd.0011156)
Supplement: S5 Table — To prevent multicollinearity, only variables that were significantly associated with in-hospital death when adjusted for long health itinerary were selected (see Table 2 & Result section) and multivariable regression was performed per delay category. (DOCX) [file pntd.0011156.s005.docx]

Supplementary Materials: Health itinerary-related survival of children under-five with severe malaria or bloodstream infection, DR Congo

Bieke Tack ^1,2,3^, Daniel Vita ^4^, José Nketo ^5^, Naomie Wasolua ^4^, Nathalie Ndengila ^4^, Natacha Herssens ^1^, Emmanuel Ntangu ^4^, Grace Kasidiko ^4^, Gaëlle Nkoji-Tunda ^6,7^, Marie-France Phoba ^6,7^, Justin Im ^8^, Hyon Jin Jeon ^8,9^, Florian Marks ^8-12^, Jaan Toelen ^3,9^, Octavie Lunguya ^6,7^ and Jan Jacobs ^1,2^

**Affiliations:**

1. Department of Clinical Sciences, Institute of Tropical Medicine, Antwerp, Belgium
2. Department of Microbiology, Immunology and Transplantation, KU Leuven, Leuven, Belgium
3. Department of Pediatrics, University Hospitals UZ Leuven, Leuven, Belgium
4. Hôpital Général de Référence Saint Luc de Kisantu, Kisantu, Democratic Republic of the Congo
5. Zone de Santé Kisantu, Kisantu, Democratic Republic of the Congo
6. Department of Microbiology, Institut National de Recherche Biomédicale, Kinshasa, Democratic Republic of the Congo
7. Department of Medical Biology, University Teaching Hospital of Kinshasa, Kinshasa, Democratic Republic of the Congo
8. International Vaccine Institute, Seoul, Republic of Korea
9. Cambridge Institute of Therapeutic Immunology and Infectious Disease, University of Cambridge School of Clinical Medicine, Cambridge, UK
10. Heidelberg Institute of Global Health, University of Heidelberg, Heidelberg, Germany
11. Madagascar Institute for Vaccine Research, University of Antananarivo, Antananarivo, Madagascar
12. Department of Development and Regeneration, KU Leuven, 3000 Leuven, Belgium

**Corresponding author:**

Bieke Tack, [btack@itg.be](mailto:btack@itg.be)

**Supplementary Table S5.** Multivariable logistic regression to assess the association between in-hospital death and sociodemographic characteristics or factors from the three-delay model (healthcare seeking, transport and prehospital patient management). To prevent multicollinearity, only variables that were significantly associated with in-hospital death when adjusted for long health itinerary were selected (see Table 2 & result section) and multivariable regression was performed per delay category.

| **Association with long health itinerary** *n = 784* | **Odds ratio  (95% CI)** | **p-value** |  |  |
| --- | --- | --- | --- | --- |
| **Multivariable logistic regression 1: Sociodemographic factors** | | | |  |
| Long health itinerary | 2.13 (1.22 – 3.72) | **0.008** |  |  |
| Age in months | 0.96 (0.93 – 0.98) | **<0.001** |  |  |
| Residence in a rural village | 1.96 (1.01 – 3.78) | 0.05 |  |  |
| **Multivariable logistic regression 2: Health care seeking behaviour** | | | | |
| Long health itinerary | 1.76 (1.00 – 3.11) | 0.05 |  |  |
| Visited a traditional practitioner | 1.72 (0.98 – 3.04) | 0.06 |  |  |
| Visited a private health centre | 1.84 (0.93 – 3.65) | 0.08 |  |  |
| Visited a health post | 4.94 (2.20 – 11.12) | **<0.001** |  |  |
| **Multivariable logistic regression 3: Prehospital patient management** | | | |  |
| Long health itinerary | 1.58 (0.89 – 2.80) | 0.12 |  |  |
| Blood transfusion | 1.33 (0.50 – 3.55) | 0.57 |  |  |
| Fluid therapy | 1.43 (0.55 – 3.72) | 0.47 |  |  |
| Systemic traditional care | 1.60 (0.86 – 2.96) | 0.14 |  |  |
| Intravenous treatment | 1.13 (0.47 -2.74) | 0.79 |  |  |
| Overnight stay | 2.19 (1.02 – 4.69) | 0.04 |  |  |
