# Supplementary material for: Health itinerary-related survival of children under-five with severe malaria or bloodstream infection, DR Congo
Source: PLoS Negl Trop Dis. 2023 Mar 6;17(3):e0011156. doi: 10.1371/journal.pntd.0011156 (PMC10019685; doi:10.1371/journal.pntd.0011156)
Supplement: S1 Document — (PDF) [file pntd.0011156.s006.pdf]

HIT Donnees generales

Numéro dans etude ________________________________

Confirmez le numéro d'étude ________________________________

Sélectez votre nom
- ◯ Adèle Zomba Lutumba
- ◯ Emmanuel Ntangu Bamikina
- ◯ Grace Kasidikoko
- ◯ Irene Kimbembi Mansosa
- ◯ Japhet Ngina Mbala
- ◯ Naomi Wasolua
- ◯ Naomie Nama Mukenyi
- ◯ Nathalie Ndengila
- ◯ Thomas Nsema Mbaki

CONSENTEMENT ÉCLAIRÉ

Consentement éclairé signé par le parent/représentant légal ou témoin (dans le cas d'un parent/représentant légal analphabète) ?
- ◯ Oui
- ◯ Non

Si le consentement éclairé n'est pas signé, l'enfant ne peut pas être inclus dans l'étude et aucune donnée ne peut être collectée !

Date consentement éclairé ________________________________

Le signataire accepte que les données d'étude soient également utilisées pour des recherches ultérieures ?
- ◯ Oui
- ◯ Non

CRITÈRES D'ÉLIGIBILITÉ

Y-a-t-il présence de fièvre actuelle (T > 37.5°C mesuré axillaire ou tympanique) ou hypothermie (< 35.5°C) au moment de l'admission ?
- ◯ Oui
- ◯ Non

Y-a-t-il présence d'une histoire de fièvre les 48 heures passées ?
- ◯ Oui
- ◯ Non

L'enfant répond aux critères pour le prélèvement d'une hémoculture (signes de sévérité) ?
- ◯ Oui
- ◯ Non

Une hémoculture a été prélevée ?
- ◯ Oui, à HSLK
- ◯ Oui, au centre de santé
- ◯ Non

Si l'enfant n'est pas eligible, l'enfant ne peut pas être inclus dans l'étude et aucune donnée ne peut être collectée !

INCLUSION DANS L'ETUDE

Date d'inclusion	______________________
Date du questionnaire	______________________
Questionnaire pas fait	◯ Pas fait : décès<br>◯ Pas fait : autre
Si autre, préciser	______________________
Est-ce que vous, comme accompagnant de l'enfant, avez déjà participé à l'étude ?	◯ Oui<br>◯ Non<br>◯ Ne sais pas
Si oui, est-ce que c'était le même enfant que vous accompagnez aujourd'hui ou un autre ?	☐ Même enfant<br>☐ Autre enfant
Si même enfant, quel était le numéro d'étude précédente ?	______________________
Si c'était un autre enfant, quel est la relation de l'autre enfant avec l'enfant que vous accompagnez aujourd'hui ?	◯ Frère/soeur<br>◯ Cousin/cousine<br>◯ Pas de famille
C'était quand ?	◯ Aujourd'hui<br>◯ Dans le passé<br>◯ Aujourd'hui et dans le passé

ADMISSION A L'HOPITAL

Date d'admission à l'hôpital	______________________
Temps d'admission à l'hôpital	◯ Matin (= après le lever de soleil)<br>◯ Après-midi (= après 12 h)<br>◯ Soir ou nuit jusqu'à minuit (= après le coucher de soleil)<br>◯ Nuit après minuit (= avant le lever de soleil)<br>◯ Inconnu

DONNEES DE L'ENFANT

Quel est le sexe de l'enfant ?	◯ Masulin<br>◯ Feminin
Date de naissance connue ?	◯ Oui<br>◯ Non
Date de naissance	______________________
Age en mois connue ?	◯ Oui<br>◯ Non

Age en mois

____
(mois)

Age en années

____
(années)

HIT Socio-demographiques

Sélectez votre nom	◯ Adèle Zomba Lutumba<br>◯ Emmanuel Ntangu Bamikina<br>◯ Grace Kasidikoko<br>◯ Irene Kimbembi Mansosa<br>◯ Japhet Ngina Mbala<br>◯ Naomi Wasolua<br>◯ Naomie Nama Mukenyi<br>◯ Nathalie Ndengila<br>◯ Thomas Nsema Mbaki

MUTUALITÉ

L'enfant est associé à la mutualité ?	◯ Oui<br>◯ Non<br>◯ Je ne sais pas

DONNEES DU SOIGNANT

Quel est votre relation avec l'enfant que vous accompagnez ?	◯ Mère<br>◯ Père<br>◯ Grand-mère<br>◯ Grand-père<br>◯ Soeur<br>◯ Frère<br>◯ Tante<br>◯ Oncle<br>◯ Cousine<br>◯ Cousin<br>◯ Autre
Autre, préciser	__________________________
Si vous n'êtes pas le parent de l'enfant, êtes-vous une représentant culturellement acceptable de l'enfant ?	◯ Oui<br>◯ Non
Vous avez quel âge ?	__________________________<br>(années)

DOMICILE DE L'ENFANT

Dans quel village/commune habite l'enfant ?	__________________________

Dans quelle aire de santé se situe le village ?

- ◯ Kavuaya
- ◯ Kikonka
- ◯ Kimuisi
- ◯ Kinkoko
- ◯ Kintanu 1
- ◯ Kintanu 2
- ◯ Kipako
- ◯ Kipasa
- ◯ Kivuangi
- ◯ Lemfu
- ◯ Madimba
- ◯ Kilenda
- ◯ Ngeba
- ◯ Nkandu
- ◯ Nzeza Nlandu
- ◯ Gare
- ◯ Yimbi
- ◯ Autre
- ◯ Je ne sais pas

Autre, préciser

____

AUTRES HABITANTS DANS LA MAISON

Hormi l'enfant malade, combien d'autres personnes vivent dans la maison ?
Enregistrez la relation avec l'enfant (père, mère, frère, etc.) et l'âge ci-dessous - 20 maximum

____

Autre habitant #1

Relation avec l'enfant

- ◯ Mère
- ◯ Père
- ◯ Grand-mère
- ◯ Grand-père
- ◯ Soeur
- ◯ Frère
- ◯ Tante
- ◯ Oncle
- ◯ Cousine
- ◯ Cousin
- ◯ Autre

Age

- ◯ < 2 ans
- ◯ >= 2 ans et < 5 ans
- ◯ >=5 ans - < 15 ans
- ◯ Adulte

Autre habitant #2

Relation avec l'enfant

- ◯ Mère
- ◯ Père
- ◯ Grand-mère
- ◯ Grand-père
- ◯ Soeur
- ◯ Frère
- ◯ Tante
- ◯ Oncle
- ◯ Cousine
- ◯ Cousin
- ◯ Autre

Age

- ◯ < 2 ans
- ◯ >= 2 ans et < 5 ans
- ◯ >=5 ans - < 15 ans
- ◯ Adulte

Autre habitant #3

Relation avec l'enfant

- ◯ Mère
- ◯ Père
- ◯ Grand-mère
- ◯ Grand-père
- ◯ Soeur
- ◯ Frère
- ◯ Tante
- ◯ Oncle
- ◯ Cousine
- ◯ Cousin
- ◯ Autre

Age

- ◯ < 2 ans
- ◯ >= 2 ans et < 5 ans
- ◯ >=5 ans - < 15 ans
- ◯ Adulte

Autre habitant #4

Relation avec l'enfant

- ◯ Mère
- ◯ Père
- ◯ Grand-mère
- ◯ Grand-père
- ◯ Soeur
- ◯ Frère
- ◯ Tante
- ◯ Oncle
- ◯ Cousine
- ◯ Cousin
- ◯ Autre

Age

- ◯ < 2 ans
- ◯ >= 2 ans et < 5 ans
- ◯ >=5 ans - < 15 ans
- ◯ Adulte

Autre habitant #5

Relation avec l'enfant	◯ Mère<br>◯ Père<br>◯ Grand-mère<br>◯ Grand-père<br>◯ Soeur<br>◯ Frère<br>◯ Tante<br>◯ Oncle<br>◯ Cousine<br>◯ Cousin<br>◯ Autre
Age	◯ < 2 ans<br>◯ >= 2 ans et < 5 ans<br>◯ >=5 ans - < 15 ans<br>◯ Adulte

Autre habitant #6

Relation avec l'enfant	◯ Mère<br>◯ Père<br>◯ Grand-mère<br>◯ Grand-père<br>◯ Soeur<br>◯ Frère<br>◯ Tante<br>◯ Oncle<br>◯ Cousine<br>◯ Cousin<br>◯ Autre
Age	◯ < 2 ans<br>◯ >= 2 ans et < 5 ans<br>◯ >=5 ans - < 15 ans<br>◯ Adulte

Autre habitant #7

Relation avec l'enfant	◯ Mère<br>◯ Père<br>◯ Grand-mère<br>◯ Grand-père<br>◯ Soeur<br>◯ Frère<br>◯ Tante<br>◯ Oncle<br>◯ Cousine<br>◯ Cousin<br>◯ Autre
Age	◯ < 2 ans<br>◯ >= 2 ans et < 5 ans<br>◯ >=5 ans - < 15 ans<br>◯ Adulte

Autre habitant #8

Relation avec l'enfant

- ◯ Mère
- ◯ Père
- ◯ Grand-mère
- ◯ Grand-père
- ◯ Soeur
- ◯ Frère
- ◯ Tante
- ◯ Oncle
- ◯ Cousine
- ◯ Cousin
- ◯ Autre

Age

- ◯ < 2 ans
- ◯ >= 2 ans et < 5 ans
- ◯ >=5 ans - < 15 ans
- ◯ Adulte

Autre habitant #9

Relation avec l'enfant

- ◯ Mère
- ◯ Père
- ◯ Grand-mère
- ◯ Grand-père
- ◯ Soeur
- ◯ Frère
- ◯ Tante
- ◯ Oncle
- ◯ Cousine
- ◯ Cousin
- ◯ Autre

Age

- ◯ < 2 ans
- ◯ >= 2 ans et < 5 ans
- ◯ >=5 ans - < 15 ans
- ◯ Adulte

Autre habitant #10

Relation avec l'enfant

- ◯ Mère
- ◯ Père
- ◯ Grand-mère
- ◯ Grand-père
- ◯ Soeur
- ◯ Frère
- ◯ Tante
- ◯ Oncle
- ◯ Cousine
- ◯ Cousin
- ◯ Autre

Age

- ◯ < 2 ans
- ◯ >= 2 ans et < 5 ans
- ◯ >=5 ans - < 15 ans
- ◯ Adulte

Autre habitant #11

Relation avec l'enfant

- ◯ Mère
- ◯ Père
- ◯ Grand-mère
- ◯ Grand-père
- ◯ Soeur
- ◯ Frère
- ◯ Tante
- ◯ Oncle
- ◯ Cousine
- ◯ Cousin
- ◯ Autre

Age

- ◯ < 2 ans
- ◯ >= 2 ans et < 5 ans
- ◯ >=5 ans - < 15 ans
- ◯ Adulte

Autre habitant #12

Relation avec l'enfant

- ◯ Mère
- ◯ Père
- ◯ Grand-mère
- ◯ Grand-père
- ◯ Soeur
- ◯ Frère
- ◯ Tante
- ◯ Oncle
- ◯ Cousine
- ◯ Cousin
- ◯ Autre

Age

- ◯ < 2 ans
- ◯ >= 2 ans et < 5 ans
- ◯ >=5 ans - < 15 ans
- ◯ Adulte

Autre habitant #13

Relation avec l'enfant

- ◯ Mère
- ◯ Père
- ◯ Grand-mère
- ◯ Grand-père
- ◯ Soeur
- ◯ Frère
- ◯ Tante
- ◯ Oncle
- ◯ Cousine
- ◯ Cousin
- ◯ Autre

Age

- ◯ < 2 ans
- ◯ >= 2 ans et < 5 ans
- ◯ >=5 ans - < 15 ans
- ◯ Adulte

Autre habitant #14

Relation avec l'enfant	◯ Mère<br>◯ Père<br>◯ Grand-mère<br>◯ Grand-père<br>◯ Soeur<br>◯ Frère<br>◯ Tante<br>◯ Oncle<br>◯ Cousine<br>◯ Cousin<br>◯ Autre
Age	◯ < 2 ans<br>◯ >= 2 ans et < 5 ans<br>◯ >=5 ans - < 15 ans<br>◯ Adulte

Autre habitant #15

Relation avec l'enfant	◯ Mère<br>◯ Père<br>◯ Grand-mère<br>◯ Grand-père<br>◯ Soeur<br>◯ Frère<br>◯ Tante<br>◯ Oncle<br>◯ Cousine<br>◯ Cousin<br>◯ Autre
Age	◯ < 2 ans<br>◯ >= 2 ans et < 5 ans<br>◯ >=5 ans - < 15 ans<br>◯ Adulte

Autre habitant #16

Relation avec l'enfant	◯ Mère<br>◯ Père<br>◯ Grand-mère<br>◯ Grand-père<br>◯ Soeur<br>◯ Frère<br>◯ Tante<br>◯ Oncle<br>◯ Cousine<br>◯ Cousin<br>◯ Autre
Age	◯ < 2 ans<br>◯ >= 2 ans et < 5 ans<br>◯ >=5 ans - < 15 ans<br>◯ Adulte

Autre habitant #17

Relation avec l'enfant

- ◯ Mère
- ◯ Père
- ◯ Grand-mère
- ◯ Grand-père
- ◯ Soeur
- ◯ Frère
- ◯ Tante
- ◯ Oncle
- ◯ Cousine
- ◯ Cousin
- ◯ Autre

Age

- ◯ < 2 ans
- ◯ >= 2 ans et < 5 ans
- ◯ >=5 ans - < 15 ans
- ◯ Adulte

Autre habitant #18

Relation avec l'enfant

- ◯ Mère
- ◯ Père
- ◯ Grand-mère
- ◯ Grand-père
- ◯ Soeur
- ◯ Frère
- ◯ Tante
- ◯ Oncle
- ◯ Cousine
- ◯ Cousin
- ◯ Autre

Age

- ◯ < 2 ans
- ◯ >= 2 ans et < 5 ans
- ◯ >=5 ans - < 15 ans
- ◯ Adulte

Autre habitant #19

Relation avec l'enfant

- ◯ Mère
- ◯ Père
- ◯ Grand-mère
- ◯ Grand-père
- ◯ Soeur
- ◯ Frère
- ◯ Tante
- ◯ Oncle
- ◯ Cousine
- ◯ Cousin
- ◯ Autre

Age

- ◯ < 2 ans
- ◯ >= 2 ans et < 5 ans
- ◯ >=5 ans - < 15 ans
- ◯ Adulte

Autre habitant #20

Relation avec l'enfant	◯ Mère<br>◯ Père<br>◯ Grand-mère<br>◯ Grand-père<br>◯ Soeur<br>◯ Frère<br>◯ Tante<br>◯ Oncle<br>◯ Cousine<br>◯ Cousin<br>◯ Autre
Age	◯ < 2 ans<br>◯ >= 2 ans et < 5 ans<br>◯ >=5 ans - < 15 ans<br>◯ Adulte

HIT Trajet pour venir a l'hopital

Sélectez votre nom	◯ Adèle Zomba Lutumba<br>◯ Emmanuel Ntangu Bamikina<br>◯ Grace Kasidikoko<br>◯ Irene Kimbembi Mansosa<br>◯ Japhet Ngina Mbala<br>◯ Naomi Wasolua<br>◯ Naomie Nama Mukenyi<br>◯ Nathalie Ndengila<br>◯ Thomas Nsema Mbaki

RÉFÉRENCE À L'HÔPITAL

Enfant référé à l'hôpital ?	◯ Oui<br>◯ Non
Référé(e) par centre de santé officielle ?	◯ Oui<br>◯ Non, centre de santé privé<br>◯ Non, centre de santé hors zone<br>◯ Non, autre prestataire
Autre, préciser	__________________________________
Quelle centre de santé vous a référé ici ?	◯ Nkandu 1<br>◯ Nkandu 2<br>◯ Kasa Vubu<br>◯ Ndinga Mbote<br>◯ Nkandu 3<br>◯ Gare<br>◯ Snel/Inkisi<br>◯ Kikonka 1<br>◯ Kikonka 2<br>◯ Kitanu/Etat<br>◯ Cerphytoco<br>◯ Omeco<br>◯ Wete<br>◯ Ngeba<br>◯ Kavuaya<br>◯ St. Pierre Boko<br>◯ Kimuisi<br>◯ Kinkoko<br>◯ Kipako<br>◯ Kituengi<br>◯ Kipasa<br>◯ Kivuangi<br>◯ Lemfu<br>◯ Madimba/Etat<br>◯ Cederi<br>◯ Kilenda<br>◯ Yimbi<br>◯ Pides<br>◯ Autre
Autre, préciser	__________________________________

Est-ce que vous savez quand vous été référé à l'hôpital ?	◯ Oui<br>◯ Non
Quand est-ce qu' ils vous ont référés à l'hôpital ?<br>Cliquez sur le calendrier et sélectionnez la date correcte	________________________
Est-ce que c'était le matin (= après le lever de soleil), l'après-midi (= après 12 h) ou le soir/la nuit (= après le coucher de soleil) ?	◯ Matin (= après le lever de soleil)<br>◯ Après-midi (= après 12 h)<br>◯ Soir ou nuit jusqu'à minuit (= après le coucher de soleil)<br>◯ Nuit après minuit (= avant le lever de soleil)<br>◯ Ne sais plus
Référence sur demande d'accompagnant ?	◯ Oui<br>◯ Non

TRANSPORT

Quand est-ce que vous avez décidé de venir à l'hôpital ?<br>Cliquez sur le calendrier et sélectionnez la date correcte	________________________
Est-ce que c'était le matin (= après le lever de soleil), l'après-midi (= après 12 h) ou le soir/la nuit (= après le coucher de soleil) ?	◯ Matin (= après le lever de soleil)<br>◯ Après-midi (= après 12 h)<br>◯ Soir ou nuit jusqu'à minuit (= après le coucher de soleil)<br>◯ Nuit après minuit (= avant le lever de soleil)<br>◯ Ne sais plus
Quand avez-vous quité pour venir à l'hôpital ?<br>Cliquez sur le calendrier et sélectionnez la date correcte	________________________
Est-ce que c'était le matin (= après le lever de soleil), l'après-midi (= après 12 h) ou le soir/la nuit (= après le coucher de soleil) ?	◯ Matin (= après le lever de soleil)<br>◯ Après-midi (= après 12 h)<br>◯ Soir ou nuit jusqu'à minuit (= après le coucher de soleil)<br>◯ Nuit après minuit (= avant le lever de soleil)<br>◯ Ne sais plus
D'oû êtes-vous partis pour venir à l'hôpital ?	◯ Centre de santé qui a référé l'enfant<br>◯ Maison<br>◯ Pharmacie privée<br>◯ Centre de santé privé<br>◯ Tradipraticien<br>◯ Prestataire privé<br>◯ Poste de santé<br>◯ Relais communautaire<br>◯ Autre (à spécifier)
Autre, spécifiez	________________________

Ça vous a pris combien de temps pour venir à l'hôpital après votre départ ?	◯ < 1 heure<br>◯ 1 - 2 heures<br>◯ 2 - 4 heures<br>◯ > 4 heures
Comment vous êtes venus ici ?	◯ A pied<br>◯ Vélo<br>◯ Moto personelle<br>◯ Mototaxi<br>◯ Voiture personelle<br>◯ Taxi<br>◯ Ambulance
Pourquoi vous avez choisi cette manière de venir ?	☐ A cause de la distance<br>☐ De l'état de la route<br>☐ De la fiabilité<br>☐ Des coûts<br>☐ A cause de l'état de l'enfant<br>☐ A cause du nombre de personne<br>☐ Absence d'autre mode de transport<br>☐ Autre<br>(Cochez toutes les réponses applicables)
Autre, préciser :	__________________________________

HIT Debut de fievre

Sélectez votre nom	◯ Adèle Zomba Lutumba<br>◯ Emmanuel Ntangu Bamikina<br>◯ Grace Kasidikoko<br>◯ Irene Kimbembi Mansosa<br>◯ Japhet Ngina Mbala<br>◯ Naomi Wasolua<br>◯ Naomie Nama Mukenyi<br>◯ Nathalie Ndengila<br>◯ Thomas Nsema Mbaki

DÉBUT DU FIÈVRE

Quand est-ce que la fièvre a commencé ?<br>Cliquez sur le calendrier et sélectionnez la date correcte	______________________
Date de début de fièvre inconnu	◯ Inconnu
Est-ce que c'était le matin, l'après-midi ou le soir/la nuit?	◯ Matin (= après le lever de soleil)<br>◯ Après-midi (= après 12 h)<br>◯ Soir ou nuit jusqu'à minuit (= après le coucher de soleil)<br>◯ Nuit après minuit (= avant le lever de soleil)<br>◯ Ne sais plus
L'enfant, a-t-il été examiné ou a-t-il reçu des soins/médicaments quelque part d'autre qu'à l'hôpital depuis le début de la fièvre ?<br>Si oui, remplissez le formulaire "Médicaments à domicle" et/ou "Trajet de soins"	◯ Oui<br>◯ Non<br>◯ Ne sais pas

HIT Medicaments a domicile

Sélectez votre nom	◯ Adèle Zomba Lutumba<br>◯ Emmanuel Ntangu Bamikina<br>◯ Grace Kasidikoko<br>◯ Irene Kimbembi Mansosa<br>◯ Japhet Ngina Mbala<br>◯ Naomi Wasolua<br>◯ Naomie Nama Mukenyi<br>◯ Nathalie Ndengila<br>◯ Thomas Nsema Mbaki

MÉDICAMENTS À DOMICILE

Est-ce que vous avez donné un traitement avec des médicaments que vous aviez encore à la maison ?	◯ Oui<br>◯ Non
Combien de traitements (que vous vous souvenez) ?	______________________
Nombre de traitements inconnu :	◯ Je ne sais pas
Combien de traitements connus (classe) que vous vous souvenez ?	______________________

Produit #1

Classe de produit	◯ Traitement traditionnel<br>◯ Antipaludéen<br>◯ Antibiotique<br>◯ Antipyrétique<br>◯ Solution de réhydratation orale<br>◯ Sérum (glucosé/fysiologique/Ringer Lactate/...)<br>◯ Transfusion sanguine<br>◯ Fer<br>◯ Vitamines/acide folique<br>◯ Combinaison de fer et vitamines/acide folique<br>◯ Vermicide<br>◯ Autre<br>◯ Inconnu
Nom du produit connu ?	◯ Oui<br>◯ Non
Nom du produit	______________________
Quand avez-vous commencé le traitement ?<br>Cliquez sur le calendrier et sélectionnez la date correcte	______________________
Est-ce que c'était le matin, l'après-midi ou le soir/la nuit ?	◯ Matin (= après le lever de soleil)<br>◯ Après-midi (= après 12 h)<br>◯ Soir ou nuit jusqu'à minuit (= après le coucher de soleil)<br>◯ Nuit après minuit (= avant le lever de soleil)<br>◯ Ne sais plus

Avez-vous arrete le traitement ?	◯ Oui<br>◯ Non
Quand avez-vous arrêté le traitement ?<br>Cliquez sur le calendrier et sélectionnez la date correcte	__________________________
Est-ce que c'était le matin, l'après-midi ou le soir/la nuit ?	◯ Matin (= après le lever de soleil)<br>◯ Après-midi (= après 12 h)<br>◯ Soir ou nuit jusqu'à minuit (= après le coucher de soleil)<br>◯ Nuit après minuit (= avant le lever de soleil)<br>◯ Ne sais plus
Voie d'administration	◯ Application locale<br>◯ Voie orale<br>◯ Intrarectal<br>◯ Injection intramusculaire<br>◯ Injection intraveineuse<br>◯ Application locale + orale<br>◯ Autre<br>◯ Inconnu
Autre, préciser	__________________________
Il s'agit d'un traitement encore en cours pour une maladie précédente ?	◯ Oui<br>◯ Non

Produit #2

Classe de produit	◯ Traitement traditionnel<br>◯ Antipaludéen<br>◯ Antibiotique<br>◯ Antipyrétique<br>◯ Solution de réhydratation orale<br>◯ Sérum (glucosé/fysiologique/Ringer Lactate/...)<br>◯ Transfusion sanguine<br>◯ Fer<br>◯ Vitamines/acide folique<br>◯ Combinaison de fer et vitamines/acide folique<br>◯ Vermicide<br>◯ Autre<br>◯ Inconnu
Nom du produit connu ?	◯ Oui<br>◯ Non
Nom du produit	__________________________
Quand avez-vous commencé le traitement ?<br>Cliquez sur le calendrier et sélectionnez la date correcte	__________________________

Est-ce que c'était le matin, l'après-midi ou le soir/la nuit ?	◯ Matin (= après le lever de soleil)<br>◯ Après-midi (= après 12 h)<br>◯ Soir ou nuit jusqu'à minuit (= après le coucher de soleil)<br>◯ Nuit après minuit (= avant le lever de soleil)<br>◯ Ne sais plus
Avez-vous arrete le traitement ?	◯ Oui<br>◯ Non
Quand avez-vous arrêté le traitement ?<br>Cliquez sur le calendrier et sélectionnez la date correcte	__________________________________
Est-ce que c'était le matin, l'après-midi ou le soir/la nuit ?	◯ Matin (= après le lever de soleil)<br>◯ Après-midi (= après 12 h)<br>◯ Soir ou nuit jusqu'à minuit (= après le coucher de soleil)<br>◯ Nuit après minuit (= avant le lever de soleil)<br>◯ Ne sais plus
Voie d'administration	◯ Application locale<br>◯ Voie orale<br>◯ Intrarectal<br>◯ Injection intramusculaire<br>◯ Injection intraveineuse<br>◯ Application locale + orale<br>◯ Autre<br>◯ Inconnu
Autre, préciser	__________________________________
Il s'agit d'un traitement encore en cours pour une maladie précédente ?	◯ Oui<br>◯ Non

Produit #3

Classe de produit	◯ Traitement traditionnel<br>◯ Antipaludéen<br>◯ Antibiotique<br>◯ Antipyrétique<br>◯ Solution de réhydratation orale<br>◯ Sérum (glucosé/fysiologique/Ringer Lactate/...)<br>◯ Transfusion sanguine<br>◯ Fer<br>◯ Vitamines/acide folique<br>◯ Combinaison de fer et vitamines/acide folique<br>◯ Vermicide<br>◯ Autre<br>◯ Inconnu
Nom du produit connu ?	◯ Oui<br>◯ Non
Nom du produit	__________________________________

Quand avez-vous commencé le traitement ?<br>Cliquez sur le calendrier et sélectionnez la date correcte	__________________________________
Est-ce que c'était le matin, l'après-midi ou le soir/la nuit ?	◯ Matin (= après le lever de soleil)<br>◯ Après-midi (= après 12 h)<br>◯ Soir ou nuit jusqu'à minuit (= après le coucher de soleil)<br>◯ Nuit après minuit (= avant le lever de soleil)<br>◯ Ne sais plus
Avez-vous arrete le traitement ?	◯ Oui<br>◯ Non
Quand avez-vous arrêté le traitement ?<br>Cliquez sur le calendrier et sélectionnez la date correcte	__________________________________
Est-ce que c'était le matin, l'après-midi ou le soir/la nuit ?	◯ Matin (= après le lever de soleil)<br>◯ Après-midi (= après 12 h)<br>◯ Soir ou nuit jusqu'à minuit (= après le coucher de soleil)<br>◯ Nuit après minuit (= avant le lever de soleil)<br>◯ Ne sais plus
Voie d'administration	◯ Application locale<br>◯ Voie orale<br>◯ Intrarectal<br>◯ Injection intramusculaire<br>◯ Injection intraveineuse<br>◯ Application locale + orale<br>◯ Autre<br>◯ Inconnu
Autre, préciser	__________________________________
Il s'agit d'un traitement encore en cours pour une maladie précédente ?	◯ Oui<br>◯ Non

Produit #4

Classe de produit	◯ Traitement traditionnel<br>◯ Antipaludéen<br>◯ Antibiotique<br>◯ Antipyrétique<br>◯ Solution de réhydratation orale<br>◯ Sérum (glucosé/fysiologique/Ringer Lactate/...)<br>◯ Transfusion sanguine<br>◯ Fer<br>◯ Vitamines/acide folique<br>◯ Combinaison de fer et vitamines/acide folique<br>◯ Vermicide<br>◯ Autre<br>◯ Inconnu
Nom du produit connu ?	◯ Oui<br>◯ Non

Nom du produit	__________________________________
Quand avez-vous commencé le traitement ?<br>Cliquez sur le calendrier et sélectionnez la date correcte	__________________________________
Est-ce que c'était le matin, l'après-midi ou le soir/la nuit ?	◯ Matin (= après le lever de soleil)<br>◯ Après-midi (= après 12 h)<br>◯ Soir ou nuit jusqu'à minuit (= après le coucher de soleil)<br>◯ Nuit après minuit (= avant le lever de soleil)<br>◯ Ne sais plus
Avez-vous arrete le traitement ?	◯ Oui<br>◯ Non
Quand avez-vous arrêté le traitement ?<br>Cliquez sur le calendrier et sélectionnez la date correcte	__________________________________
Est-ce que c'était le matin, l'après-midi ou le soir/la nuit ?	◯ Matin (= après le lever de soleil)<br>◯ Après-midi (= après 12 h)<br>◯ Soir ou nuit jusqu'à minuit (= après le coucher de soleil)<br>◯ Nuit après minuit (= avant le lever de soleil)<br>◯ Ne sais plus
Voie d'administration	◯ Application locale<br>◯ Voie orale<br>◯ Intrarectal<br>◯ Injection intramusculaire<br>◯ Injection intraveineuse<br>◯ Application locale + orale<br>◯ Autre<br>◯ Inconnu
Autre, préciser	__________________________________
Il s'agit d'un traitement encore en cours pour une maladie précédente ?	◯ Oui<br>◯ Non

Produit #5

Classe de produit	◯ Traitement traditionnel<br>◯ Antipaludéen<br>◯ Antibiotique<br>◯ Antipyrétique<br>◯ Solution de réhydratation orale<br>◯ Sérum (glucosé/fysiologique/Ringer Lactate/...)<br>◯ Transfusion sanguine<br>◯ Fer<br>◯ Vitamines/acide folique<br>◯ Combinaison de fer et vitamines/acide folique<br>◯ Vermicide<br>◯ Autre<br>◯ Inconnu

Nom du produit connu ?	◯ Oui<br>◯ Non
Nom du produit	__________________________________
Quand avez-vous commencé le traitement ?<br>Cliquez sur le calendrier et sélectionnez la date correcte	__________________________________
Est-ce que c'était le matin, l'après-midi ou le soir/la nuit ?	◯ Matin (= après le lever de soleil)<br>◯ Après-midi (= après 12 h)<br>◯ Soir ou nuit jusqu'à minuit (= après le coucher de soleil)<br>◯ Nuit après minuit (= avant le lever de soleil)<br>◯ Ne sais plus
Avez-vous arrete le traitement ?	◯ Oui<br>◯ Non
Quand avez-vous arrêté le traitement ?<br>Cliquez sur le calendrier et sélectionnez la date correcte	__________________________________
Est-ce que c'était le matin, l'après-midi ou le soir/la nuit ?	◯ Matin (= après le lever de soleil)<br>◯ Après-midi (= après 12 h)<br>◯ Soir ou nuit jusqu'à minuit (= après le coucher de soleil)<br>◯ Nuit après minuit (= avant le lever de soleil)<br>◯ Ne sais plus
Voie d'administration	◯ Application locale<br>◯ Voie orale<br>◯ Intrarectal<br>◯ Injection intramusculaire<br>◯ Injection intraveineuse<br>◯ Application locale + orale<br>◯ Autre<br>◯ Inconnu
Autre, préciser	__________________________________
Il s'agit d'un traitement encore en cours pour une maladie précédente ?	◯ Oui<br>◯ Non

HIT Trajet de soins 1

Sélectez votre nom	◯ Adèle Zomba Lutumba<br>◯ Emmanuel Ntangu Bamikina<br>◯ Grace Kasidikoko<br>◯ Irene Kimbembi Mansosa<br>◯ Japhet Ngina Mbala<br>◯ Naomi Wasolua<br>◯ Naomie Nama Mukenyi<br>◯ Nathalie Ndengila<br>◯ Thomas Nsema Mbaki

TRAJET DE SOINS DE SANTÉ

Est-ce que vous avez consulté d'autres prestataires de santé ?	◯ Oui<br>◯ Non
Combien de fois avez-vous consulté un prestataire de santé avant de venir à l'hôpital (nombre de visites que vous vous souvenez) ?	______________________

Remplissez un formulaire "Trajet de soins" pour chaque visite à chaque prestataire de santé que vous avez consulté.

Le prestataire était le quantième prestataire que vous avez consulté ?	◯ 1e<br>◯ 2e<br>◯ 3e<br>◯ 4e<br>◯ 5e
S'agit-il d'une nouvelle visite, mais d'un prestataire pour lequel vous avez déjà rempli un trajet de soins ?	◯ Non<br>◯ Oui, même que trajet de soins 1<br>◯ Oui, même que trajet de soins 2<br>◯ Oui, même que trajet de soins 3<br>◯ Oui, même que trajet de soins 4<br>◯ Oui, même que trajet de soins 5
Qui avez-vous consulté ou qui a donné les médicaments ?	◯ Tradipraticien (bokoko/nganga/féticheur/église)<br>◯ Pharmacie privée ou vendeur des médicaments<br>◯ Relais communautaire<br>◯ Poste de santé<br>◯ Centre de santé privé<br>◯ Centre de santé des structures de référence<br>◯ Prestataire privé<br>◯ Je ne sais pas
Est-ce que c'est ce prestataire qui vous a référé à l'hôpital ?	◯ Oui, pendant cette visite<br>◯ Oui, mais pendant une visite suivante<br>◯ Non
Quand avez-vous fait ça ?<br>Cliquez sur le calendrier et sélectionnez la date correcte	______________________
Si la date est inconnu	◯ Je ne sais pas

Est-ce que c'était le matin, l'après-midi ou le soir/la nuit ?	◯ Matin (= après le lever de soleil)<br>◯ Après-midi (= après 12 h)<br>◯ Soir ou nuit jusqu'à minuit (= après le coucher de soleil)<br>◯ Nuit après minuit (= avant le lever de soleil)<br>◯ Je ne sais pas
Avez-vous passer la nuit là-bas ?	◯ Oui<br>◯ Non
Si oui, jusqu'à quand ?	__________________________________
Jusqu'à le matin, l'après-midi ou le soir/la nuit ?	◯ Matin (= après le lever de soleil)<br>◯ Après-midi (= après 12 h)<br>◯ Soir ou nuit jusqu'à minuit (= après le coucher de soleil)<br>◯ Nuit après minuit (= avant le lever de soleil)<br>◯ Je ne sais pas
Est-ce que vous avez dû vous déplacer en dehors de votre domicile ?	◯ Oui, mais nous sommes restés dans notre village<br>◯ Oui, autre village du même aire de santé<br>◯ Oui, autre aire de santé<br>◯ Non (resté à domicile)<br>◯ Je ne sais pas
Si vous avez dû vous déplacer, comment vous êtes allées là-bas ?	◯ A pied<br>◯ Vélo<br>◯ Moto personelle<br>◯ Mototaxi<br>◯ Voiture personelle<br>◯ Taxi<br>◯ Ambulance<br>◯ Je ne sais pas
Si autre, préciser :	__________________________________
Pourquoi vous avez choisi cette manière d'y aller ou choisi de rester à domicile ?	☐ A cause de la distance<br>☐ De l'état de la route<br>☐ De la fiabilité<br>☐ Des coûts<br>☐ A cause de l'état de l'enfant<br>☐ A cause du nombre de personne<br>☐ Absence d'autre mode de transport<br>☐ Autre<br>☐ Je ne sais pas<br>(Cochez toutes les réponses applicables)
Autre, préciser :	__________________________________
Est-ce qu'ils ont prélevé du sang de l'enfant pour faire des tests laboratoires ?	◯ Oui<br>◯ Non<br>◯ Je ne sais pas
Est-ce qu'ils ont donné un traitement à l'enfant ?	◯ Oui<br>◯ Non<br>◯ Je ne sais pas

Combien de traitements ?

____

Nombre de traitements inconnu : ◯ Je ne sais pas

Combien de traitements connu (classe) ?

____

Produit #1

Classe de produit
◯ Traitement traditionnel
◯ Antipaludéen
◯ Antibiotique
◯ Antipyrétique
◯ Solution de réhydratation orale
◯ Sérum (glucosé/fysiologique/Ringer Lactate/...)
◯ Transfusion sanguine
◯ Fer
◯ Vitamines/acide folique
◯ Combinaison de fer et vitamines/acide folique
◯ Vermicide
◯ Autre
◯ Inconnu

Nom du produit connu ?
◯ Oui
◯ Non

Nom du produit

____

Voie d'administration
◯ Application locale
◯ Voie orale
◯ Intrarectal
◯ Injection intramusculaire
◯ Injection intraveineuse
◯ Application locale + orale
◯ Autre
◯ Inconnu

Autre, préciser

____

Traitement déjà arreté ?
◯ Oui
◯ Non
◯ Je ne sais pas

Date d'arrêt

____

Temps d'arrêt
◯ Matin (= après le lever de soleil)
◯ Après-midi (= après 12 h)
◯ Soir ou nuit jusqu'à minuit (= après le coucher de soleil)
◯ Nuit après minuit (= avant le lever de soleil)
◯ Je ne sais pas

Produit #2

Classe de produit	◯ Traitement traditionnel<br>◯ Antipaludéen<br>◯ Antibiotique<br>◯ Antipyrétique<br>◯ Solution de réhydratation orale<br>◯ Sérum (glucosé/fysiologique/Ringer Lactate/...)<br>◯ Transfusion sanguine<br>◯ Fer<br>◯ Vitamines/acide folique<br>◯ Combinaison de fer et vitamines/acide folique<br>◯ Vermicide<br>◯ Autre<br>◯ Inconnu
Nom du produit connu ?	◯ Oui<br>◯ Non
Nom du produit	______________________
Voie d'administration	◯ Application locale<br>◯ Voie orale<br>◯ Intrarectal<br>◯ Injection intramusculaire<br>◯ Injection intraveineuse<br>◯ Application locale + orale<br>◯ Autre<br>◯ Inconnu
Autre, préciser	______________________
Traitement déjà arreté ?	◯ Oui<br>◯ Non<br>◯ Je ne sais pas
Date d'arrêt	______________________
Temps d'arrêt	◯ Matin (= après le lever de soleil)<br>◯ Après-midi (= après 12 h)<br>◯ Soir ou nuit jusqu'à minuit (= après le coucher de soleil)<br>◯ Nuit après minuit (= avant le lever de soleil)<br>◯ Je ne sais pas

Produit #3

Classe de produit	◯ Traitement traditionnel<br>◯ Antipaludéen<br>◯ Antibiotique<br>◯ Antipyrétique<br>◯ Solution de réhydratation orale<br>◯ Sérum (glucosé/fysiologique/Ringer Lactate/...)<br>◯ Transfusion sanguine<br>◯ Fer<br>◯ Vitamines/acide folique<br>◯ Combinaison de fer et vitamines/acide folique<br>◯ Vermicide<br>◯ Autre<br>◯ Inconnu
Nom du produit connu ?	◯ Oui<br>◯ Non
Nom du produit	______________________
Voie d'administration	◯ Application locale<br>◯ Voie orale<br>◯ Intrarectal<br>◯ Injection intramusculaire<br>◯ Injection intraveineuse<br>◯ Application locale + orale<br>◯ Autre<br>◯ Inconnu
Autre, préciser	______________________
Traitement déjà arreté ?	◯ Oui<br>◯ Non<br>◯ Je ne sais pas
Date d'arrêt	______________________
Temps d'arrêt	◯ Matin (= après le lever de soleil)<br>◯ Après-midi (= après 12 h)<br>◯ Soir ou nuit jusqu'à minuit (= après le coucher de soleil)<br>◯ Nuit après minuit (= avant le lever de soleil)<br>◯ Je ne sais pas

Produit #4

Classe de produit	◯ Traitement traditionnel<br>◯ Antipaludéen<br>◯ Antibiotique<br>◯ Antipyrétique<br>◯ Solution de réhydratation orale<br>◯ Sérum (glucosé/fysiologique/Ringer Lactate/...)<br>◯ Transfusion sanguine<br>◯ Fer<br>◯ Vitamines/acide folique<br>◯ Combinaison de fer et vitamines/acide folique<br>◯ Vermicide<br>◯ Autre<br>◯ Inconnu
Nom du produit connu ?	◯ Oui<br>◯ Non
Nom du produit	______________________
Voie d'administration	◯ Application locale<br>◯ Voie orale<br>◯ Intrarectal<br>◯ Injection intramusculaire<br>◯ Injection intraveineuse<br>◯ Application locale + orale<br>◯ Autre<br>◯ Inconnu
Autre, préciser	______________________
Traitement déjà arreté ?	◯ Oui<br>◯ Non<br>◯ Je ne sais pas
Date d'arrêt	______________________
Temps d'arrêt	◯ Matin (= après le lever de soleil)<br>◯ Après-midi (= après 12 h)<br>◯ Soir ou nuit jusqu'à minuit (= après le coucher de soleil)<br>◯ Nuit après minuit (= avant le lever de soleil)<br>◯ Je ne sais pas

Produit #5

Classe de produit	◯ Traitement traditionnel<br>◯ Antipaludéen<br>◯ Antibiotique<br>◯ Antipyrétique<br>◯ Solution de réhydratation orale<br>◯ Sérum (glucosé/fysiologique/Ringer Lactate/...)<br>◯ Transfusion sanguine<br>◯ Fer<br>◯ Vitamines/acide folique<br>◯ Combinaison de fer et vitamines/acide folique<br>◯ Vermicide<br>◯ Autre<br>◯ Inconnu
Nom du produit connu ?	◯ Oui<br>◯ Non
Nom du produit	__________________________________
Voie d'administration	◯ Application locale<br>◯ Voie orale<br>◯ Intrarectal<br>◯ Injection intramusculaire<br>◯ Injection intraveineuse<br>◯ Application locale + orale<br>◯ Autre<br>◯ Inconnu
Autre, préciser	__________________________________
Traitement déjà arreté ?	◯ Oui<br>◯ Non<br>◯ Je ne sais pas
Date d'arrêt	__________________________________
Temps d'arrêt	◯ Matin (= après le lever de soleil)<br>◯ Après-midi (= après 12 h)<br>◯ Soir ou nuit jusqu'à minuit (= après le coucher de soleil)<br>◯ Nuit après minuit (= avant le lever de soleil)<br>◯ Je ne sais pas

Produit #6

Classe de produit	◯ Traitement traditionnel<br>◯ Antipaludéen<br>◯ Antibiotique<br>◯ Antipyrétique<br>◯ Solution de réhydratation orale<br>◯ Sérum (glucosé/fysiologique/Ringer Lactate/...)<br>◯ Transfusion sanguine<br>◯ Fer<br>◯ Vitamines/acide folique<br>◯ Combinaison de fer et vitamines/acide folique<br>◯ Vermicide<br>◯ Autre<br>◯ Inconnu
Nom du produit connu ?	◯ Oui<br>◯ Non
Nom du produit	__________________________
Voie d'administration	◯ Application locale<br>◯ Voie orale<br>◯ Intrarectal<br>◯ Injection intramusculaire<br>◯ Injection intraveineuse<br>◯ Application locale + orale<br>◯ Autre<br>◯ Inconnu
Autre, préciser	__________________________
Traitement déjà arreté ?	◯ Oui<br>◯ Non<br>◯ Je ne sais pas
Date d'arrêt	__________________________
Temps d'arrêt	◯ Matin (= après le lever de soleil)<br>◯ Après-midi (= après 12 h)<br>◯ Soir ou nuit jusqu'à minuit (= après le coucher de soleil)<br>◯ Nuit après minuit (= avant le lever de soleil)<br>◯ Je ne sais pas

Produit #7

Classe de produit	◯ Traitement traditionnel<br>◯ Antipaludéen<br>◯ Antibiotique<br>◯ Antipyrétique<br>◯ Solution de réhydratation orale<br>◯ Sérum (glucosé/fysiologique/Ringer Lactate/...)<br>◯ Transfusion sanguine<br>◯ Fer<br>◯ Vitamines/acide folique<br>◯ Combinaison de fer et vitamines/acide folique<br>◯ Vermicide<br>◯ Autre<br>◯ Inconnu
Nom du produit connu ?	◯ Oui<br>◯ Non
Nom du produit	__________________________
Voie d'administration	◯ Application locale<br>◯ Voie orale<br>◯ Intrarectal<br>◯ Injection intramusculaire<br>◯ Injection intraveineuse<br>◯ Application locale + orale<br>◯ Autre<br>◯ Inconnu
Autre, préciser	__________________________
Traitement déjà arreté ?	◯ Oui<br>◯ Non<br>◯ Je ne sais pas
Date d'arrêt	__________________________
Temps d'arrêt	◯ Matin (= après le lever de soleil)<br>◯ Après-midi (= après 12 h)<br>◯ Soir ou nuit jusqu'à minuit (= après le coucher de soleil)<br>◯ Nuit après minuit (= avant le lever de soleil)<br>◯ Je ne sais pas

Produit #8

Classe de produit	◯ Traitement traditionnel<br>◯ Antipaludéen<br>◯ Antibiotique<br>◯ Antipyrétique<br>◯ Solution de réhydratation orale<br>◯ Sérum (glucosé/fysiologique/Ringer Lactate/...)<br>◯ Transfusion sanguine<br>◯ Fer<br>◯ Vitamines/acide folique<br>◯ Combinaison de fer et vitamines/acide folique<br>◯ Vermicide<br>◯ Autre<br>◯ Inconnu
Nom du produit connu ?	◯ Oui<br>◯ Non
Nom du produit	______________________
Voie d'administration	◯ Application locale<br>◯ Voie orale<br>◯ Intrarectal<br>◯ Injection intramusculaire<br>◯ Injection intraveineuse<br>◯ Application locale + orale<br>◯ Autre<br>◯ Inconnu
Autre, préciser	______________________
Traitement déjà arreté ?	◯ Oui<br>◯ Non<br>◯ Je ne sais pas
Date d'arrêt	______________________
Temps d'arrêt	◯ Matin (= après le lever de soleil)<br>◯ Après-midi (= après 12 h)<br>◯ Soir ou nuit jusqu'à minuit (= après le coucher de soleil)<br>◯ Nuit après minuit (= avant le lever de soleil)<br>◯ Je ne sais pas

Produit #9

Classe de produit	◯ Traitement traditionnel<br>◯ Antipaludéen<br>◯ Antibiotique<br>◯ Antipyrétique<br>◯ Solution de réhydratation orale<br>◯ Sérum (glucosé/fysiologique/Ringer Lactate/...)<br>◯ Transfusion sanguine<br>◯ Fer<br>◯ Vitamines/acide folique<br>◯ Combinaison de fer et vitamines/acide folique<br>◯ Vermicide<br>◯ Autre<br>◯ Inconnu
Nom du produit connu ?	◯ Oui<br>◯ Non
Nom du produit	__________________________________
Voie d'administration	◯ Application locale<br>◯ Voie orale<br>◯ Intrarectal<br>◯ Injection intramusculaire<br>◯ Injection intraveineuse<br>◯ Application locale + orale<br>◯ Autre<br>◯ Inconnu
Autre, préciser	__________________________________
Traitement déjà arreté ?	◯ Oui<br>◯ Non<br>◯ Je ne sais pas
Date d'arrêt	__________________________________
Temps d'arrêt	◯ Matin (= après le lever de soleil)<br>◯ Après-midi (= après 12 h)<br>◯ Soir ou nuit jusqu'à minuit (= après le coucher de soleil)<br>◯ Nuit après minuit (= avant le lever de soleil)<br>◯ Je ne sais pas

Produit #10

Classe de produit	◯ Traitement traditionnel<br>◯ Antipaludéen<br>◯ Antibiotique<br>◯ Antipyrétique<br>◯ Solution de réhydratation orale<br>◯ Sérum (glucosé/fysiologique/Ringer Lactate/...)<br>◯ Transfusion sanguine<br>◯ Fer<br>◯ Vitamines/acide folique<br>◯ Combinaison de fer et vitamines/acide folique<br>◯ Vermicide<br>◯ Autre<br>◯ Inconnu
Nom du produit connu ?	◯ Oui<br>◯ Non
Nom du produit	______________________
Voie d'administration	◯ Application locale<br>◯ Voie orale<br>◯ Intrarectal<br>◯ Injection intramusculaire<br>◯ Injection intraveineuse<br>◯ Application locale + orale<br>◯ Autre<br>◯ Inconnu
Autre, préciser	______________________
Traitement déjà arreté ?	◯ Oui<br>◯ Non<br>◯ Je ne sais pas
Date d'arrêt	______________________
Temps d'arrêt	◯ Matin (= après le lever de soleil)<br>◯ Après-midi (= après 12 h)<br>◯ Soir ou nuit jusqu'à minuit (= après le coucher de soleil)<br>◯ Nuit après minuit (= avant le lever de soleil)<br>◯ Je ne sais pas

HIT Trajet de soins 2

TRAJET DE SOINS DE SANTÉ #2	
Le prestataire était le quantième prestataire que vous avez consulté ?	◯ 1e<br>◯ 2e<br>◯ 3e<br>◯ 4e<br>◯ 5e
S'agit-il d'une nouvelle visite, mais d'un prestataire pour lequel vous avez déjà rempli un trajet de soins ?	◯ Non<br>◯ Oui, même que trajet de soins 1<br>◯ Oui, même que trajet de soins 2<br>◯ Oui, même que trajet de soins 3<br>◯ Oui, même que trajet de soins 4<br>◯ Oui, même que trajet de soins 5
Qui avez-vous consulté ou qui a donné les médicaments ?	◯ Tradipraticien (bokoko/nganga/féticheur/église)<br>◯ Pharmacie privée ou vendeur des médicaments<br>◯ Relais communautaire<br>◯ Poste de santé<br>◯ Centre de santé privé<br>◯ Centre de santé des structures de référence<br>◯ Prestataire privé<br>◯ Je ne sais pas
Est-ce que c'est ce prestataire qui vous a référé à l'hôpital ?	◯ Oui, pendant cette visite<br>◯ Oui, mais pendant une visite suivante<br>◯ Non
Quand avez-vous fait ça ?<br>Cliquez sur le calendrier et sélectionnez la date correcte	______________________________
Si la date est inconnu	◯ Je ne sais pas
Est-ce que c'était le matin, l'après-midi ou le soir/la nuit ?	◯ Matin (= après le lever de soleil)<br>◯ Après-midi (= après 12 h)<br>◯ Soir ou nuit jusqu'à minuit (= après le coucher de soleil)<br>◯ Nuit après minuit (= avant le lever de soleil)<br>◯ Je ne sais pas
Avez-vous passer la nuit là-bas ?	◯ Oui<br>◯ Non
Si oui, jusqu'à quand ?	______________________________
Jusqu'à le matin, l'après-midi ou le soir/la nuit ?	◯ Matin (= après le lever de soleil)<br>◯ Après-midi (= après 12 h)<br>◯ Soir ou nuit jusqu'à minuit (= après le coucher de soleil)<br>◯ Nuit après minuit (= avant le lever de soleil)<br>◯ Je ne sais pas

Est-ce que vous avez dû vous déplacer en dehors de votre village ou aire de santé ?	◯ Oui, mais nous sommes restés dans notre village<br>◯ Oui, autre village du même aire de santé<br>◯ Oui, autre aire de santé<br>◯ Non (resté à domicile)<br>◯ Je ne sais pas
Si vous avez dû vous déplacer, comment vous êtes allées là-bas ?	◯ A pied<br>◯ Vélo<br>◯ Moto personelle<br>◯ Mototaxi<br>◯ Voiture personelle<br>◯ Taxi<br>◯ Ambulance<br>◯ Je ne sais pas
Si autre, préciser :	______________________________
Pourquoi vous avez choisi cette manière d'y aller ou choisi de rester à domicile ?	☐ A cause de la distance<br>☐ De l'état de la route<br>☐ De la fiabilité<br>☐ Des coûts<br>☐ A cause de l'état de l'enfant<br>☐ A cause du nombre de personne<br>☐ Absence d'autre mode de transport<br>☐ Autre<br>☐ Je ne sais pas<br>(Cochez toutes les réponses applicables)
Autre, préciser :	______________________________
Est-ce qu'ils ont prélevé du sang de l'enfant pour faire des tests laboratoires ?	◯ Oui<br>◯ Non<br>◯ Je ne sais pas
Est-ce qu'ils ont donné un traitement à l'enfant ?	◯ Oui<br>◯ Non<br>◯ Je ne sais pas
Combien de traitements ?	______________________________
Nombre de traitements inconnu :	◯ Je ne sais pas
Combien de traitements connu (classe et nom de produit, voie d'administration) ?<br>10 max.	______________________________

Produit #1

Classe de produit	◯ Traitement traditionnel<br>◯ Antipaludéen<br>◯ Antibiotique<br>◯ Antipyrétique<br>◯ Solution de réhydratation orale<br>◯ Sérum (glucosé/fysiologique/Ringer Lactate/...)<br>◯ Transfusion sanguine<br>◯ Fer<br>◯ Vitamines/acide folique<br>◯ Combinaison de fer et vitamines/acide folique<br>◯ Vermicide<br>◯ Autre<br>◯ Inconnu
Nom du produit connu ?	◯ Oui<br>◯ Non
Nom du produit	______________________
Voie d'administration	◯ Application locale<br>◯ Voie orale<br>◯ Intrarectal<br>◯ Injection intramusculaire<br>◯ Injection intraveineuse<br>◯ Application locale + orale<br>◯ Autre<br>◯ Inconnu
Autre, préciser	______________________
Traitement déjà arreté ?	◯ Oui<br>◯ Non<br>◯ Je ne sais pas
Date d'arrêt	______________________
Temps d'arrêt	◯ Matin (= après le lever de soleil)<br>◯ Après-midi (= après 12 h)<br>◯ Soir ou nuit jusqu'à minuit (= après le coucher de soleil)<br>◯ Nuit après minuit (= avant le lever de soleil)<br>◯ Je ne sais pas

Produit #2

Classe de produit	◯ Traitement traditionnel<br>◯ Antipaludéen<br>◯ Antibiotique<br>◯ Antipyrétique<br>◯ Solution de réhydratation orale<br>◯ Sérum (glucosé/fysiologique/Ringer Lactate/...)<br>◯ Transfusion sanguine<br>◯ Fer<br>◯ Vitamines/acide folique<br>◯ Combinaison de fer et vitamines/acide folique<br>◯ Vermicide<br>◯ Autre<br>◯ Inconnu
Nom du produit connu ?	◯ Oui<br>◯ Non
Nom du produit	______________________
Voie d'administration	◯ Application locale<br>◯ Voie orale<br>◯ Intrarectal<br>◯ Injection intramusculaire<br>◯ Injection intraveineuse<br>◯ Application locale + orale<br>◯ Autre<br>◯ Inconnu
Autre, préciser	______________________
Traitement déjà arreté ?	◯ Oui<br>◯ Non<br>◯ Je ne sais pas
Date d'arrêt	______________________
Temps d'arrêt	◯ Matin (= après le lever de soleil)<br>◯ Après-midi (= après 12 h)<br>◯ Soir ou nuit jusqu'à minuit (= après le coucher de soleil)<br>◯ Nuit après minuit (= avant le lever de soleil)<br>◯ Je ne sais pas

Produit #3

Classe de produit	◯ Traitement traditionnel<br>◯ Antipaludéen<br>◯ Antibiotique<br>◯ Antipyrétique<br>◯ Solution de réhydratation orale<br>◯ Sérum (glucosé/fysiologique/Ringer Lactate/...)<br>◯ Transfusion sanguine<br>◯ Fer<br>◯ Vitamines/acide folique<br>◯ Combinaison de fer et vitamines/acide folique<br>◯ Vermicide<br>◯ Autre<br>◯ Inconnu
Nom du produit connu ?	◯ Oui<br>◯ Non
Nom du produit	______________________________
Voie d'administration	◯ Application locale<br>◯ Voie orale<br>◯ Intrarectal<br>◯ Injection intramusculaire<br>◯ Injection intraveineuse<br>◯ Application locale + orale<br>◯ Autre<br>◯ Inconnu
Autre, préciser	______________________________
Traitement déjà arreté ?	◯ Oui<br>◯ Non<br>◯ Je ne sais pas
Date d'arrêt	______________________________
Temps d'arrêt	◯ Matin (= après le lever de soleil)<br>◯ Après-midi (= après 12 h)<br>◯ Soir ou nuit jusqu'à minuit (= après le coucher de soleil)<br>◯ Nuit après minuit (= avant le lever de soleil)<br>◯ Je ne sais pas

Produit #4

Classe de produit	◯ Traitement traditionnel<br>◯ Antipaludéen<br>◯ Antibiotique<br>◯ Antipyrétique<br>◯ Solution de réhydratation orale<br>◯ Sérum (glucosé/fysiologique/Ringer Lactate/...)<br>◯ Transfusion sanguine<br>◯ Fer<br>◯ Vitamines/acide folique<br>◯ Combinaison de fer et vitamines/acide folique<br>◯ Vermicide<br>◯ Autre<br>◯ Inconnu
Nom du produit connu ?	◯ Oui<br>◯ Non
Nom du produit	______________________
Voie d'administration	◯ Application locale<br>◯ Voie orale<br>◯ Intrarectal<br>◯ Injection intramusculaire<br>◯ Injection intraveineuse<br>◯ Application locale + orale<br>◯ Autre<br>◯ Inconnu
Autre, préciser	______________________
Traitement déjà arreté ?	◯ Oui<br>◯ Non<br>◯ Je ne sais pas
Date d'arrêt	______________________
Temps d'arrêt	◯ Matin (= après le lever de soleil)<br>◯ Après-midi (= après 12 h)<br>◯ Soir ou nuit jusqu'à minuit (= après le coucher de soleil)<br>◯ Nuit après minuit (= avant le lever de soleil)<br>◯ Je ne sais pas

Produit #5

Classe de produit	◯ Traitement traditionnel<br>◯ Antipaludéen<br>◯ Antibiotique<br>◯ Antipyrétique<br>◯ Solution de réhydratation orale<br>◯ Sérum (glucosé/fysiologique/Ringer Lactate/...)<br>◯ Transfusion sanguine<br>◯ Fer<br>◯ Vitamines/acide folique<br>◯ Combinaison de fer et vitamines/acide folique<br>◯ Vermicide<br>◯ Autre<br>◯ Inconnu
Nom du produit connu ?	◯ Oui<br>◯ Non
Nom du produit	__________________________
Voie d'administration	◯ Application locale<br>◯ Voie orale<br>◯ Intrarectal<br>◯ Injection intramusculaire<br>◯ Injection intraveineuse<br>◯ Application locale + orale<br>◯ Autre<br>◯ Inconnu
Autre, préciser	__________________________
Traitement déjà arreté ?	◯ Oui<br>◯ Non<br>◯ Je ne sais pas
Date d'arrêt	__________________________
Temps d'arrêt	◯ Matin (= après le lever de soleil)<br>◯ Après-midi (= après 12 h)<br>◯ Soir ou nuit jusqu'à minuit (= après le coucher de soleil)<br>◯ Nuit après minuit (= avant le lever de soleil)<br>◯ Je ne sais pas

Produit #6

Classe de produit	◯ Traitement traditionnel<br>◯ Antipaludéen<br>◯ Antibiotique<br>◯ Antipyrétique<br>◯ Solution de réhydratation orale<br>◯ Sérum (glucosé/fysiologique/Ringer Lactate/...)<br>◯ Transfusion sanguine<br>◯ Fer<br>◯ Vitamines/acide folique<br>◯ Combinaison de fer et vitamines/acide folique<br>◯ Vermicide<br>◯ Autre<br>◯ Inconnu
Nom du produit connu ?	◯ Oui<br>◯ Non
Nom du produit	__________________________________
Voie d'administration	◯ Application locale<br>◯ Voie orale<br>◯ Intrarectal<br>◯ Injection intramusculaire<br>◯ Injection intraveineuse<br>◯ Application locale + orale<br>◯ Autre<br>◯ Inconnu
Autre, préciser	__________________________________
Traitement déjà arreté ?	◯ Oui<br>◯ Non<br>◯ Je ne sais pas
Date d'arrêt	__________________________________
Temps d'arrêt	◯ Matin (= après le lever de soleil)<br>◯ Après-midi (= après 12 h)<br>◯ Soir ou nuit jusqu'à minuit (= après le coucher de soleil)<br>◯ Nuit après minuit (= avant le lever de soleil)<br>◯ Je ne sais pas

Produit #7

Classe de produit	◯ Traitement traditionnel<br>◯ Antipaludéen<br>◯ Antibiotique<br>◯ Antipyrétique<br>◯ Solution de réhydratation orale<br>◯ Sérum (glucosé/fysiologique/Ringer Lactate/...)<br>◯ Transfusion sanguine<br>◯ Fer<br>◯ Vitamines/acide folique<br>◯ Combinaison de fer et vitamines/acide folique<br>◯ Vermicide<br>◯ Autre<br>◯ Inconnu
Nom du produit connu ?	◯ Oui<br>◯ Non
Nom du produit	__________________________
Voie d'administration	◯ Application locale<br>◯ Voie orale<br>◯ Intrarectal<br>◯ Injection intramusculaire<br>◯ Injection intraveineuse<br>◯ Application locale + orale<br>◯ Autre<br>◯ Inconnu
Autre, préciser	__________________________
Traitement déjà arreté ?	◯ Oui<br>◯ Non<br>◯ Je ne sais pas
Date d'arrêt	__________________________
Temps d'arrêt	◯ Matin (= après le lever de soleil)<br>◯ Après-midi (= après 12 h)<br>◯ Soir ou nuit jusqu'à minuit (= après le coucher de soleil)<br>◯ Nuit après minuit (= avant le lever de soleil)<br>◯ Je ne sais pas

Produit #8

Classe de produit	◯ Traitement traditionnel<br>◯ Antipaludéen<br>◯ Antibiotique<br>◯ Antipyrétique<br>◯ Solution de réhydratation orale<br>◯ Sérum (glucosé/fysiologique/Ringer Lactate/...)<br>◯ Transfusion sanguine<br>◯ Fer<br>◯ Vitamines/acide folique<br>◯ Combinaison de fer et vitamines/acide folique<br>◯ Vermicide<br>◯ Autre<br>◯ Inconnu
Nom du produit connu ?	◯ Oui<br>◯ Non
Nom du produit	__________________________________
Voie d'administration	◯ Application locale<br>◯ Voie orale<br>◯ Intrarectal<br>◯ Injection intramusculaire<br>◯ Injection intraveineuse<br>◯ Application locale + orale<br>◯ Autre<br>◯ Inconnu
Autre, préciser	__________________________________
Traitement déjà arreté ?	◯ Oui<br>◯ Non<br>◯ Je ne sais pas
Date d'arrêt	__________________________________
Temps d'arrêt	◯ Matin (= après le lever de soleil)<br>◯ Après-midi (= après 12 h)<br>◯ Soir ou nuit jusqu'à minuit (= après le coucher de soleil)<br>◯ Nuit après minuit (= avant le lever de soleil)<br>◯ Je ne sais pas

Produit #9

Classe de produit	◯ Traitement traditionnel<br>◯ Antipaludéen<br>◯ Antibiotique<br>◯ Antipyrétique<br>◯ Solution de réhydratation orale<br>◯ Sérum (glucosé/fysiologique/Ringer Lactate/...)<br>◯ Transfusion sanguine<br>◯ Fer<br>◯ Vitamines/acide folique<br>◯ Combinaison de fer et vitamines/acide folique<br>◯ Vermicide<br>◯ Autre<br>◯ Inconnu
Nom du produit connu ?	◯ Oui<br>◯ Non
Nom du produit	________________________
Voie d'administration	◯ Application locale<br>◯ Voie orale<br>◯ Intrarectal<br>◯ Injection intramusculaire<br>◯ Injection intraveineuse<br>◯ Application locale + orale<br>◯ Autre<br>◯ Inconnu
Autre, préciser	________________________
Traitement déjà arreté ?	◯ Oui<br>◯ Non<br>◯ Je ne sais pas
Date d'arrêt	________________________
Temps d'arrêt	◯ Matin (= après le lever de soleil)<br>◯ Après-midi (= après 12 h)<br>◯ Soir ou nuit jusqu'à minuit (= après le coucher de soleil)<br>◯ Nuit après minuit (= avant le lever de soleil)<br>◯ Je ne sais pas

Produit #10

Classe de produit	◯ Traitement traditionnel<br>◯ Antipaludéen<br>◯ Antibiotique<br>◯ Antipyrétique<br>◯ Solution de réhydratation orale<br>◯ Sérum (glucosé/fysiologique/Ringer Lactate/...)<br>◯ Transfusion sanguine<br>◯ Fer<br>◯ Vitamines/acide folique<br>◯ Combinaison de fer et vitamines/acide folique<br>◯ Vermicide<br>◯ Autre<br>◯ Inconnu
Nom du produit connu ?	◯ Oui<br>◯ Non
Nom du produit	______________________
Voie d'administration	◯ Application locale<br>◯ Voie orale<br>◯ Intrarectal<br>◯ Injection intramusculaire<br>◯ Injection intraveineuse<br>◯ Application locale + orale<br>◯ Autre<br>◯ Inconnu
Autre, préciser	______________________
Traitement déjà arreté ?	◯ Oui<br>◯ Non<br>◯ Je ne sais pas
Date d'arrêt	______________________
Temps d'arrêt	◯ Matin (= après le lever de soleil)<br>◯ Après-midi (= après 12 h)<br>◯ Soir ou nuit jusqu'à minuit (= après le coucher de soleil)<br>◯ Nuit après minuit (= avant le lever de soleil)<br>◯ Je ne sais pas

HIT Trajet de soins 3

TRAJET DE SOINS DE SANTÉ #3

Le prestataire était le quantième prestataire que vous avez consulté ?	◯ 1e<br>◯ 2e<br>◯ 3e<br>◯ 4e<br>◯ 5e
S'agit-il d'une nouvelle visite, mais d'un prestataire pour lequel vous avez déjà rempli un trajet de soins ?	◯ Non<br>◯ Oui, même que trajet de soins 1<br>◯ Oui, même que trajet de soins 2<br>◯ Oui, même que trajet de soins 3<br>◯ Oui, même que trajet de soins 4<br>◯ Oui, même que trajet de soins 5
Qui avez-vous consulté ou qui a donné les médicaments ?	◯ Tradipraticien (bokoko/nganga/féticheur/église)<br>◯ Pharmacie privée ou vendeur des médicaments<br>◯ Relais communautaire<br>◯ Poste de santé<br>◯ Centre de santé privé<br>◯ Centre de santé des structures de référence<br>◯ Prestataire privé<br>◯ Je ne sais pas
Est-ce que c'est ce prestataire qui vous a référé à l'hôpital ?	◯ Oui, pendant cette visite<br>◯ Oui, mais pendant une visite suivante<br>◯ Non
Quand avez-vous fait ça ?<br>Cliquez sur le calendrier et sélectionnez la date correcte	______________________________
Si la date est inconnu	◯ Je ne sais pas
Est-ce que c'était le matin, l'après-midi ou le soir/la nuit ?	◯ Matin (= après le lever de soleil)<br>◯ Après-midi (= après 12 h)<br>◯ Soir ou nuit jusqu'à minuit (= après le coucher de soleil)<br>◯ Nuit après minuit (= avant le lever de soleil)<br>◯ Je ne sais pas
Avez-vous passer la nuit là-bas ?	◯ Oui<br>◯ Non
Si oui, jusqu'à quand ?	______________________________
Jusqu'à le matin, l'après-midi ou le soir/la nuit ?	◯ Matin (= après le lever de soleil)<br>◯ Après-midi (= après 12 h)<br>◯ Soir ou nuit jusqu'à minuit (= après le coucher de soleil)<br>◯ Nuit après minuit (= avant le lever de soleil)<br>◯ Je ne sais pas

Est-ce que vous avez dû vous déplacer en dehors de votre village ou aire de santé ?	◯ Oui, mais nous sommes restés dans notre village<br>◯ Oui, autre village du même aire de santé<br>◯ Oui, autre aire de santé<br>◯ Non (resté à domicile)<br>◯ Je ne sais pas
Si vous avez dû vous déplacer, comment vous êtes allées là-bas ?	◯ A pied<br>◯ Vélo<br>◯ Moto personelle<br>◯ Mototaxi<br>◯ Voiture personelle<br>◯ Taxi<br>◯ Ambulance<br>◯ Je ne sais pas
Si autre, préciser :	______________________________
Pourquoi vous avez choisi cette manière d'y aller ou choisi de rester à domicile ?	☐ A cause de la distance<br>☐ De l'état de la route<br>☐ De la fiabilité<br>☐ Des coûts<br>☐ A cause de l'état de l'enfant<br>☐ A cause du nombre de personne<br>☐ Absence d'autre mode de transport<br>☐ Autre<br>☐ Je ne sais pas<br>(Cochez toutes les réponses applicables)
Autre, préciser :	______________________________
Est-ce qu'ils ont prélevé du sang de l'enfant pour faire des tests laboratoires ?	◯ Oui<br>◯ Non<br>◯ Je ne sais pas
Est-ce qu'ils ont donné un traitement à l'enfant ?	◯ Oui<br>◯ Non<br>◯ Je ne sais pas
Combien de traitements ?	______________________________
Nombre de traitements inconnu :	◯ Je ne sais pas
Combien de traitements connu (classe et nom de produit, voie d'administration) ?<br>10 max.	______________________________

Produit #1

Classe de produit	◯ Traitement traditionnel<br>◯ Antipaludéen<br>◯ Antibiotique<br>◯ Antipyrétique<br>◯ Solution de réhydratation orale<br>◯ Sérum (glucosé/fysiologique/Ringer Lactate/...)<br>◯ Transfusion sanguine<br>◯ Fer<br>◯ Vitamines/acide folique<br>◯ Combinaison de fer et vitamines/acide folique<br>◯ Vermicide<br>◯ Autre<br>◯ Inconnu
Nom du produit connu ?	◯ Oui<br>◯ Non
Nom du produit	__________________________________
Voie d'administration	◯ Application locale<br>◯ Voie orale<br>◯ Intrarectal<br>◯ Injection intramusculaire<br>◯ Injection intraveineuse<br>◯ Application locale + orale<br>◯ Autre<br>◯ Inconnu
Autre, préciser	__________________________________
Traitement déjà arreté ?	◯ Oui<br>◯ Non<br>◯ Je ne sais pas
Date d'arrêt	__________________________________
Temps d'arrêt	◯ Matin (= après le lever de soleil)<br>◯ Après-midi (= après 12 h)<br>◯ Soir ou nuit jusqu'à minuit (= après le coucher de soleil)<br>◯ Nuit après minuit (= avant le lever de soleil)<br>◯ Je ne sais pas

Produit #2

Classe de produit	◯ Traitement traditionnel<br>◯ Antipaludéen<br>◯ Antibiotique<br>◯ Antipyrétique<br>◯ Solution de réhydratation orale<br>◯ Sérum (glucosé/fysiologique/Ringer Lactate/...)<br>◯ Transfusion sanguine<br>◯ Fer<br>◯ Vitamines/acide folique<br>◯ Combinaison de fer et vitamines/acide folique<br>◯ Vermicide<br>◯ Autre<br>◯ Inconnu
Nom du produit connu ?	◯ Oui<br>◯ Non
Nom du produit	______________________________
Voie d'administration	◯ Application locale<br>◯ Voie orale<br>◯ Intrarectal<br>◯ Injection intramusculaire<br>◯ Injection intraveineuse<br>◯ Application locale + orale<br>◯ Autre<br>◯ Inconnu
Autre, préciser	______________________________
Traitement déjà arreté ?	◯ Oui<br>◯ Non<br>◯ Je ne sais pas
Date d'arrêt	______________________________
Temps d'arrêt	◯ Matin (= après le lever de soleil)<br>◯ Après-midi (= après 12 h)<br>◯ Soir ou nuit jusqu'à minuit (= après le coucher de soleil)<br>◯ Nuit après minuit (= avant le lever de soleil)<br>◯ Je ne sais pas

Produit #3

Classe de produit	◯ Traitement traditionnel<br>◯ Antipaludéen<br>◯ Antibiotique<br>◯ Antipyrétique<br>◯ Solution de réhydratation orale<br>◯ Sérum (glucosé/fysiologique/Ringer Lactate/...)<br>◯ Transfusion sanguine<br>◯ Fer<br>◯ Vitamines/acide folique<br>◯ Combinaison de fer et vitamines/acide folique<br>◯ Vermicide<br>◯ Autre<br>◯ Inconnu
Nom du produit connu ?	◯ Oui<br>◯ Non
Nom du produit	__________________________________
Voie d'administration	◯ Application locale<br>◯ Voie orale<br>◯ Intrarectal<br>◯ Injection intramusculaire<br>◯ Injection intraveineuse<br>◯ Application locale + orale<br>◯ Autre<br>◯ Inconnu
Autre, préciser	__________________________________
Traitement déjà arreté ?	◯ Oui<br>◯ Non<br>◯ Je ne sais pas
Date d'arrêt	__________________________________
Temps d'arrêt	◯ Matin (= après le lever de soleil)<br>◯ Après-midi (= après 12 h)<br>◯ Soir ou nuit jusqu'à minuit (= après le coucher de soleil)<br>◯ Nuit après minuit (= avant le lever de soleil)<br>◯ Je ne sais pas

Produit #4

Classe de produit	◯ Traitement traditionnel<br>◯ Antipaludéen<br>◯ Antibiotique<br>◯ Antipyrétique<br>◯ Solution de réhydratation orale<br>◯ Sérum (glucosé/fysiologique/Ringer Lactate/...)<br>◯ Transfusion sanguine<br>◯ Fer<br>◯ Vitamines/acide folique<br>◯ Combinaison de fer et vitamines/acide folique<br>◯ Vermicide<br>◯ Autre<br>◯ Inconnu
Nom du produit connu ?	◯ Oui<br>◯ Non
Nom du produit	__________________________
Voie d'administration	◯ Application locale<br>◯ Voie orale<br>◯ Intrarectal<br>◯ Injection intramusculaire<br>◯ Injection intraveineuse<br>◯ Application locale + orale<br>◯ Autre<br>◯ Inconnu
Autre, préciser	__________________________
Traitement déjà arreté ?	◯ Oui<br>◯ Non<br>◯ Je ne sais pas
Date d'arrêt	__________________________
Temps d'arrêt	◯ Matin (= après le lever de soleil)<br>◯ Après-midi (= après 12 h)<br>◯ Soir ou nuit jusqu'à minuit (= après le coucher de soleil)<br>◯ Nuit après minuit (= avant le lever de soleil)<br>◯ Je ne sais pas

Produit #5

Classe de produit	◯ Traitement traditionnel<br>◯ Antipaludéen<br>◯ Antibiotique<br>◯ Antipyrétique<br>◯ Solution de réhydratation orale<br>◯ Sérum (glucosé/fysiologique/Ringer Lactate/...)<br>◯ Transfusion sanguine<br>◯ Fer<br>◯ Vitamines/acide folique<br>◯ Combinaison de fer et vitamines/acide folique<br>◯ Vermicide<br>◯ Autre<br>◯ Inconnu
Nom du produit connu ?	◯ Oui<br>◯ Non
Nom du produit	__________________________________
Voie d'administration	◯ Application locale<br>◯ Voie orale<br>◯ Intrarectal<br>◯ Injection intramusculaire<br>◯ Injection intraveineuse<br>◯ Application locale + orale<br>◯ Autre<br>◯ Inconnu
Autre, préciser	__________________________________
Traitement déjà arreté ?	◯ Oui<br>◯ Non<br>◯ Je ne sais pas
Date d'arrêt	__________________________________
Temps d'arrêt	◯ Matin (= après le lever de soleil)<br>◯ Après-midi (= après 12 h)<br>◯ Soir ou nuit jusqu'à minuit (= après le coucher de soleil)<br>◯ Nuit après minuit (= avant le lever de soleil)<br>◯ Je ne sais pas

Produit #6

Classe de produit	◯ Traitement traditionnel<br>◯ Antipaludéen<br>◯ Antibiotique<br>◯ Antipyrétique<br>◯ Solution de réhydratation orale<br>◯ Sérum (glucosé/fysiologique/Ringer Lactate/...)<br>◯ Transfusion sanguine<br>◯ Fer<br>◯ Vitamines/acide folique<br>◯ Combinaison de fer et vitamines/acide folique<br>◯ Vermicide<br>◯ Autre<br>◯ Inconnu
Nom du produit connu ?	◯ Oui<br>◯ Non
Nom du produit	__________________________________
Voie d'administration	◯ Application locale<br>◯ Voie orale<br>◯ Intrarectal<br>◯ Injection intramusculaire<br>◯ Injection intraveineuse<br>◯ Application locale + orale<br>◯ Autre<br>◯ Inconnu
Autre, préciser	__________________________________
Traitement déjà arreté ?	◯ Oui<br>◯ Non<br>◯ Je ne sais pas
Date d'arrêt	__________________________________
Temps d'arrêt	◯ Matin (= après le lever de soleil)<br>◯ Après-midi (= après 12 h)<br>◯ Soir ou nuit jusqu'à minuit (= après le coucher de soleil)<br>◯ Nuit après minuit (= avant le lever de soleil)<br>◯ Je ne sais pas

Produit #7

Classe de produit	◯ Traitement traditionnel<br>◯ Antipaludéen<br>◯ Antibiotique<br>◯ Antipyrétique<br>◯ Solution de réhydratation orale<br>◯ Sérum (glucosé/fysiologique/Ringer Lactate/...)<br>◯ Transfusion sanguine<br>◯ Fer<br>◯ Vitamines/acide folique<br>◯ Combinaison de fer et vitamines/acide folique<br>◯ Vermicide<br>◯ Autre<br>◯ Inconnu
Nom du produit connu ?	◯ Oui<br>◯ Non
Nom du produit	__________________________________
Voie d'administration	◯ Application locale<br>◯ Voie orale<br>◯ Intrarectal<br>◯ Injection intramusculaire<br>◯ Injection intraveineuse<br>◯ Application locale + orale<br>◯ Autre<br>◯ Inconnu
Autre, préciser	__________________________________
Traitement déjà arreté ?	◯ Oui<br>◯ Non<br>◯ Je ne sais pas
Date d'arrêt	__________________________________
Temps d'arrêt	◯ Matin (= après le lever de soleil)<br>◯ Après-midi (= après 12 h)<br>◯ Soir ou nuit jusqu'à minuit (= après le coucher de soleil)<br>◯ Nuit après minuit (= avant le lever de soleil)<br>◯ Je ne sais pas

Produit #8

Classe de produit	◯ Traitement traditionnel<br>◯ Antipaludéen<br>◯ Antibiotique<br>◯ Antipyrétique<br>◯ Solution de réhydratation orale<br>◯ Sérum (glucosé/fysiologique/Ringer Lactate/...)<br>◯ Transfusion sanguine<br>◯ Fer<br>◯ Vitamines/acide folique<br>◯ Combinaison de fer et vitamines/acide folique<br>◯ Vermicide<br>◯ Autre<br>◯ Inconnu
Nom du produit connu ?	◯ Oui<br>◯ Non
Nom du produit	__________________________________
Voie d'administration	◯ Application locale<br>◯ Voie orale<br>◯ Intrarectal<br>◯ Injection intramusculaire<br>◯ Injection intraveineuse<br>◯ Application locale + orale<br>◯ Autre<br>◯ Inconnu
Autre, préciser	__________________________________
Traitement déjà arreté ?	◯ Oui<br>◯ Non<br>◯ Je ne sais pas
Date d'arrêt	__________________________________
Temps d'arrêt	◯ Matin (= après le lever de soleil)<br>◯ Après-midi (= après 12 h)<br>◯ Soir ou nuit jusqu'à minuit (= après le coucher de soleil)<br>◯ Nuit après minuit (= avant le lever de soleil)<br>◯ Je ne sais pas

Produit #9

Classe de produit	◯ Traitement traditionnel<br>◯ Antipaludéen<br>◯ Antibiotique<br>◯ Antipyrétique<br>◯ Solution de réhydratation orale<br>◯ Sérum (glucosé/fysiologique/Ringer Lactate/...)<br>◯ Transfusion sanguine<br>◯ Fer<br>◯ Vitamines/acide folique<br>◯ Combinaison de fer et vitamines/acide folique<br>◯ Vermicide<br>◯ Autre<br>◯ Inconnu
Nom du produit connu ?	◯ Oui<br>◯ Non
Nom du produit	______________________________
Voie d'administration	◯ Application locale<br>◯ Voie orale<br>◯ Intrarectal<br>◯ Injection intramusculaire<br>◯ Injection intraveineuse<br>◯ Application locale + orale<br>◯ Autre<br>◯ Inconnu
Autre, préciser	______________________________
Traitement déjà arreté ?	◯ Oui<br>◯ Non<br>◯ Je ne sais pas
Date d'arrêt	______________________________
Temps d'arrêt	◯ Matin (= après le lever de soleil)<br>◯ Après-midi (= après 12 h)<br>◯ Soir ou nuit jusqu'à minuit (= après le coucher de soleil)<br>◯ Nuit après minuit (= avant le lever de soleil)<br>◯ Je ne sais pas

Produit #10

Classe de produit	◯ Traitement traditionnel<br>◯ Antipaludéen<br>◯ Antibiotique<br>◯ Antipyrétique<br>◯ Solution de réhydratation orale<br>◯ Sérum (glucosé/fysiologique/Ringer Lactate/...)<br>◯ Transfusion sanguine<br>◯ Fer<br>◯ Vitamines/acide folique<br>◯ Combinaison de fer et vitamines/acide folique<br>◯ Vermicide<br>◯ Autre<br>◯ Inconnu
Nom du produit connu ?	◯ Oui<br>◯ Non
Nom du produit	__________________________________
Voie d'administration	◯ Application locale<br>◯ Voie orale<br>◯ Intrarectal<br>◯ Injection intramusculaire<br>◯ Injection intraveineuse<br>◯ Application locale + orale<br>◯ Autre<br>◯ Inconnu
Autre, préciser	__________________________________
Traitement déjà arreté ?	◯ Oui<br>◯ Non<br>◯ Je ne sais pas
Date d'arrêt	__________________________________
Temps d'arrêt	◯ Matin (= après le lever de soleil)<br>◯ Après-midi (= après 12 h)<br>◯ Soir ou nuit jusqu'à minuit (= après le coucher de soleil)<br>◯ Nuit après minuit (= avant le lever de soleil)<br>◯ Je ne sais pas

HIT Trajet de soins 4

TRAJET DE SOINS DE SANTÉ #4	
Le prestataire était le quantième prestataire que vous avez consulté ?	◯ 1e<br>◯ 2e<br>◯ 3e<br>◯ 4e<br>◯ 5e
S'agit-il d'une nouvelle visite, mais d'un prestataire pour lequel vous avez déjà rempli un trajet de soins ?	◯ Non<br>◯ Oui, même que trajet de soins 1<br>◯ Oui, même que trajet de soins 2<br>◯ Oui, même que trajet de soins 3<br>◯ Oui, même que trajet de soins 4<br>◯ Oui, même que trajet de soins 5
Qui avez-vous consulté ou qui a donné les médicaments ?	◯ Tradipraticien (bokoko/nganga/féticheur/église)<br>◯ Pharmacie privée ou vendeur des médicaments<br>◯ Relais communautaire<br>◯ Poste de santé<br>◯ Centre de santé privé<br>◯ Centre de santé des structures de référence<br>◯ Prestataire privé<br>◯ Je ne sais pas
Est-ce que c'est ce prestataire qui vous a référé à l'hôpital ?	◯ Oui, pendant cette visite<br>◯ Oui, mais pendant une visite suivante<br>◯ Non
Quand avez-vous fait ça ?<br>Cliquez sur le calendrier et sélectionnez la date correcte	________________________________
Si la date est inconnu	◯ Je ne sais pas
Est-ce que c'était le matin, l'après-midi ou le soir/la nuit ?	◯ Matin (= après le lever de soleil)<br>◯ Après-midi (= après 12 h)<br>◯ Soir ou nuit jusqu'à minuit (= après le coucher de soleil)<br>◯ Nuit après minuit (= avant le lever de soleil)<br>◯ Je ne sais pas
Avez-vous passer la nuit là-bas ?	◯ Oui<br>◯ Non
Si oui, jusqu'à quand ?	________________________________
Jusqu'à le matin, l'après-midi ou le soir/la nuit ?	◯ Matin (= après le lever de soleil)<br>◯ Après-midi (= après 12 h)<br>◯ Soir ou nuit jusqu'à minuit (= après le coucher de soleil)<br>◯ Nuit après minuit (= avant le lever de soleil)<br>◯ Je ne sais pas

Est-ce que vous avez dû vous déplacer en dehors de votre village ou aire de santé ?	◯ Oui, mais nous sommes restés dans notre village<br>◯ Oui, autre village du même aire de santé<br>◯ Oui, autre aire de santé<br>◯ Non (resté à domicile)<br>◯ Je ne sais pas
Si vous avez dû vous déplacer, comment vous êtes allées là-bas ?	◯ A pied<br>◯ Vélo<br>◯ Moto personelle<br>◯ Mototaxi<br>◯ Voiture personelle<br>◯ Taxi<br>◯ Ambulance<br>◯ Je ne sais pas
Si autre, préciser :	______________________________
Pourquoi vous avez choisi cette manière d'y aller ou choisi de rester à domicile ?	☐ A cause de la distance<br>☐ De l'état de la route<br>☐ De la fiabilité<br>☐ Des coûts<br>☐ A cause de l'état de l'enfant<br>☐ A cause du nombre de personne<br>☐ Absence d'autre mode de transport<br>☐ Autre<br>☐ Je ne sais pas<br>(Cochez toutes les réponses applicables)
Autre, préciser :	______________________________
Est-ce qu'ils ont prélevé du sang de l'enfant pour faire des tests laboratoires ?	◯ Oui<br>◯ Non<br>◯ Je ne sais pas
Est-ce qu'ils ont donné un traitement à l'enfant ?	◯ Oui<br>◯ Non<br>◯ Je ne sais pas
Combien de traitements ?	______________________________
Nombre de traitements inconnu :	◯ Je ne sais pas
Combien de traitements connu (classe et nom de produit, voie d'administration) ?<br>10 max.	______________________________

Produit #1

Classe de produit	◯ Traitement traditionnel<br>◯ Antipaludéen<br>◯ Antibiotique<br>◯ Antipyrétique<br>◯ Solution de réhydratation orale<br>◯ Sérum (glucosé/fysiologique/Ringer Lactate/...)<br>◯ Transfusion sanguine<br>◯ Fer<br>◯ Vitamines/acide folique<br>◯ Combinaison de fer et vitamines/acide folique<br>◯ Vermicide<br>◯ Autre<br>◯ Inconnu
Nom du produit connu ?	◯ Oui<br>◯ Non
Nom du produit	______________________
Voie d'administration	◯ Application locale<br>◯ Voie orale<br>◯ Intrarectal<br>◯ Injection intramusculaire<br>◯ Injection intraveineuse<br>◯ Application locale + orale<br>◯ Autre<br>◯ Inconnu
Autre, préciser	______________________
Traitement déjà arreté ?	◯ Oui<br>◯ Non<br>◯ Je ne sais pas
Date d'arrêt	______________________
Temps d'arrêt	◯ Matin (= après le lever de soleil)<br>◯ Après-midi (= après 12 h)<br>◯ Soir ou nuit jusqu'à minuit (= après le coucher de soleil)<br>◯ Nuit après minuit (= avant le lever de soleil)<br>◯ Je ne sais pas

Produit #2

Classe de produit	◯ Traitement traditionnel<br>◯ Antipaludéen<br>◯ Antibiotique<br>◯ Antipyrétique<br>◯ Solution de réhydratation orale<br>◯ Sérum (glucosé/fysiologique/Ringer Lactate/...)<br>◯ Transfusion sanguine<br>◯ Fer<br>◯ Vitamines/acide folique<br>◯ Combinaison de fer et vitamines/acide folique<br>◯ Vermicide<br>◯ Autre<br>◯ Inconnu
Nom du produit connu ?	◯ Oui<br>◯ Non
Nom du produit	__________________________________
Voie d'administration	◯ Application locale<br>◯ Voie orale<br>◯ Intrarectal<br>◯ Injection intramusculaire<br>◯ Injection intraveineuse<br>◯ Application locale + orale<br>◯ Autre<br>◯ Inconnu
Autre, préciser	__________________________________
Traitement déjà arreté ?	◯ Oui<br>◯ Non<br>◯ Je ne sais pas
Date d'arrêt	__________________________________
Temps d'arrêt	◯ Matin (= après le lever de soleil)<br>◯ Après-midi (= après 12 h)<br>◯ Soir ou nuit jusqu'à minuit (= après le coucher de soleil)<br>◯ Nuit après minuit (= avant le lever de soleil)<br>◯ Je ne sais pas

Produit #3

Classe de produit	◯ Traitement traditionnel<br>◯ Antipaludéen<br>◯ Antibiotique<br>◯ Antipyrétique<br>◯ Solution de réhydratation orale<br>◯ Sérum (glucosé/fysiologique/Ringer Lactate/...)<br>◯ Transfusion sanguine<br>◯ Fer<br>◯ Vitamines/acide folique<br>◯ Combinaison de fer et vitamines/acide folique<br>◯ Vermicide<br>◯ Autre<br>◯ Inconnu
Nom du produit connu ?	◯ Oui<br>◯ Non
Nom du produit	__________________________
Voie d'administration	◯ Application locale<br>◯ Voie orale<br>◯ Intrarectal<br>◯ Injection intramusculaire<br>◯ Injection intraveineuse<br>◯ Application locale + orale<br>◯ Autre<br>◯ Inconnu
Autre, préciser	__________________________
Traitement déjà arreté ?	◯ Oui<br>◯ Non<br>◯ Je ne sais pas
Date d'arrêt	__________________________
Temps d'arrêt	◯ Matin (= après le lever de soleil)<br>◯ Après-midi (= après 12 h)<br>◯ Soir ou nuit jusqu'à minuit (= après le coucher de soleil)<br>◯ Nuit après minuit (= avant le lever de soleil)<br>◯ Je ne sais pas

Produit #4

Classe de produit	◯ Traitement traditionnel<br>◯ Antipaludéen<br>◯ Antibiotique<br>◯ Antipyrétique<br>◯ Solution de réhydratation orale<br>◯ Sérum (glucosé/fysiologique/Ringer Lactate/...)<br>◯ Transfusion sanguine<br>◯ Fer<br>◯ Vitamines/acide folique<br>◯ Combinaison de fer et vitamines/acide folique<br>◯ Vermicide<br>◯ Autre<br>◯ Inconnu
Nom du produit connu ?	◯ Oui<br>◯ Non
Nom du produit	______________________________
Voie d'administration	◯ Application locale<br>◯ Voie orale<br>◯ Intrarectal<br>◯ Injection intramusculaire<br>◯ Injection intraveineuse<br>◯ Application locale + orale<br>◯ Autre<br>◯ Inconnu
Autre, préciser	______________________________
Traitement déjà arreté ?	◯ Oui<br>◯ Non<br>◯ Je ne sais pas
Date d'arrêt	______________________________
Temps d'arrêt	◯ Matin (= après le lever de soleil)<br>◯ Après-midi (= après 12 h)<br>◯ Soir ou nuit jusqu'à minuit (= après le coucher de soleil)<br>◯ Nuit après minuit (= avant le lever de soleil)<br>◯ Je ne sais pas

Produit #5

Classe de produit	◯ Traitement traditionnel<br>◯ Antipaludéen<br>◯ Antibiotique<br>◯ Antipyrétique<br>◯ Solution de réhydratation orale<br>◯ Sérum (glucosé/fysiologique/Ringer Lactate/...)<br>◯ Transfusion sanguine<br>◯ Fer<br>◯ Vitamines/acide folique<br>◯ Combinaison de fer et vitamines/acide folique<br>◯ Vermicide<br>◯ Autre<br>◯ Inconnu
Nom du produit connu ?	◯ Oui<br>◯ Non
Nom du produit	__________________________________
Voie d'administration	◯ Application locale<br>◯ Voie orale<br>◯ Intrarectal<br>◯ Injection intramusculaire<br>◯ Injection intraveineuse<br>◯ Application locale + orale<br>◯ Autre<br>◯ Inconnu
Autre, préciser	__________________________________
Traitement déjà arreté ?	◯ Oui<br>◯ Non<br>◯ Je ne sais pas
Date d'arrêt	__________________________________
Temps d'arrêt	◯ Matin (= après le lever de soleil)<br>◯ Après-midi (= après 12 h)<br>◯ Soir ou nuit jusqu'à minuit (= après le coucher de soleil)<br>◯ Nuit après minuit (= avant le lever de soleil)<br>◯ Je ne sais pas

Produit #6

Classe de produit	◯ Traitement traditionnel<br>◯ Antipaludéen<br>◯ Antibiotique<br>◯ Antipyrétique<br>◯ Solution de réhydratation orale<br>◯ Sérum (glucosé/fysiologique/Ringer Lactate/...)<br>◯ Transfusion sanguine<br>◯ Fer<br>◯ Vitamines/acide folique<br>◯ Combinaison de fer et vitamines/acide folique<br>◯ Vermicide<br>◯ Autre<br>◯ Inconnu
Nom du produit connu ?	◯ Oui<br>◯ Non
Nom du produit	__________________________
Voie d'administration	◯ Application locale<br>◯ Voie orale<br>◯ Intrarectal<br>◯ Injection intramusculaire<br>◯ Injection intraveineuse<br>◯ Application locale + orale<br>◯ Autre<br>◯ Inconnu
Autre, préciser	__________________________
Traitement déjà arreté ?	◯ Oui<br>◯ Non<br>◯ Je ne sais pas
Date d'arrêt	__________________________
Temps d'arrêt	◯ Matin (= après le lever de soleil)<br>◯ Après-midi (= après 12 h)<br>◯ Soir ou nuit jusqu'à minuit (= après le coucher de soleil)<br>◯ Nuit après minuit (= avant le lever de soleil)<br>◯ Je ne sais pas

Produit #7

Classe de produit	◯ Traitement traditionnel<br>◯ Antipaludéen<br>◯ Antibiotique<br>◯ Antipyrétique<br>◯ Solution de réhydratation orale<br>◯ Sérum (glucosé/fysiologique/Ringer Lactate/...)<br>◯ Transfusion sanguine<br>◯ Fer<br>◯ Vitamines/acide folique<br>◯ Combinaison de fer et vitamines/acide folique<br>◯ Vermicide<br>◯ Autre<br>◯ Inconnu
Nom du produit connu ?	◯ Oui<br>◯ Non
Nom du produit	__________________________________
Voie d'administration	◯ Application locale<br>◯ Voie orale<br>◯ Intrarectal<br>◯ Injection intramusculaire<br>◯ Injection intraveineuse<br>◯ Application locale + orale<br>◯ Autre<br>◯ Inconnu
Autre, préciser	__________________________________
Traitement déjà arreté ?	◯ Oui<br>◯ Non<br>◯ Je ne sais pas
Date d'arrêt	__________________________________
Temps d'arrêt	◯ Matin (= après le lever de soleil)<br>◯ Après-midi (= après 12 h)<br>◯ Soir ou nuit jusqu'à minuit (= après le coucher de soleil)<br>◯ Nuit après minuit (= avant le lever de soleil)<br>◯ Je ne sais pas

Produit #8

Classe de produit	◯ Traitement traditionnel<br>◯ Antipaludéen<br>◯ Antibiotique<br>◯ Antipyrétique<br>◯ Solution de réhydratation orale<br>◯ Sérum (glucosé/fysiologique/Ringer Lactate/...)<br>◯ Transfusion sanguine<br>◯ Fer<br>◯ Vitamines/acide folique<br>◯ Combinaison de fer et vitamines/acide folique<br>◯ Vermicide<br>◯ Autre<br>◯ Inconnu
Nom du produit connu ?	◯ Oui<br>◯ Non
Nom du produit	______________________
Voie d'administration	◯ Application locale<br>◯ Voie orale<br>◯ Intrarectal<br>◯ Injection intramusculaire<br>◯ Injection intraveineuse<br>◯ Application locale + orale<br>◯ Autre<br>◯ Inconnu
Autre, préciser	______________________
Traitement déjà arreté ?	◯ Oui<br>◯ Non<br>◯ Je ne sais pas
Date d'arrêt	______________________
Temps d'arrêt	◯ Matin (= après le lever de soleil)<br>◯ Après-midi (= après 12 h)<br>◯ Soir ou nuit jusqu'à minuit (= après le coucher de soleil)<br>◯ Nuit après minuit (= avant le lever de soleil)<br>◯ Je ne sais pas

Produit #9

Classe de produit	◯ Traitement traditionnel<br>◯ Antipaludéen<br>◯ Antibiotique<br>◯ Antipyrétique<br>◯ Solution de réhydratation orale<br>◯ Sérum (glucosé/fysiologique/Ringer Lactate/...)<br>◯ Transfusion sanguine<br>◯ Fer<br>◯ Vitamines/acide folique<br>◯ Combinaison de fer et vitamines/acide folique<br>◯ Vermicide<br>◯ Autre<br>◯ Inconnu
Nom du produit connu ?	◯ Oui<br>◯ Non
Nom du produit	__________________________
Voie d'administration	◯ Application locale<br>◯ Voie orale<br>◯ Intrarectal<br>◯ Injection intramusculaire<br>◯ Injection intraveineuse<br>◯ Application locale + orale<br>◯ Autre<br>◯ Inconnu
Autre, préciser	__________________________
Traitement déjà arreté ?	◯ Oui<br>◯ Non<br>◯ Je ne sais pas
Date d'arrêt	__________________________
Temps d'arrêt	◯ Matin (= après le lever de soleil)<br>◯ Après-midi (= après 12 h)<br>◯ Soir ou nuit jusqu'à minuit (= après le coucher de soleil)<br>◯ Nuit après minuit (= avant le lever de soleil)<br>◯ Je ne sais pas

Produit #10

Classe de produit	◯ Traitement traditionnel<br>◯ Antipaludéen<br>◯ Antibiotique<br>◯ Antipyrétique<br>◯ Solution de réhydratation orale<br>◯ Sérum (glucosé/fysiologique/Ringer Lactate/...)<br>◯ Transfusion sanguine<br>◯ Fer<br>◯ Vitamines/acide folique<br>◯ Combinaison de fer et vitamines/acide folique<br>◯ Vermicide<br>◯ Autre<br>◯ Inconnu
Nom du produit connu ?	◯ Oui<br>◯ Non
Nom du produit	__________________________________
Voie d'administration	◯ Application locale<br>◯ Voie orale<br>◯ Intrarectal<br>◯ Injection intramusculaire<br>◯ Injection intraveineuse<br>◯ Application locale + orale<br>◯ Autre<br>◯ Inconnu
Autre, préciser	__________________________________
Traitement déjà arreté ?	◯ Oui<br>◯ Non<br>◯ Je ne sais pas
Date d'arrêt	__________________________________
Temps d'arrêt	◯ Matin (= après le lever de soleil)<br>◯ Après-midi (= après 12 h)<br>◯ Soir ou nuit jusqu'à minuit (= après le coucher de soleil)<br>◯ Nuit après minuit (= avant le lever de soleil)<br>◯ Je ne sais pas

HIT Trajet de soins 5

TRAJET DE SOINS DE SANTÉ #5

Le prestataire était le quantième prestataire que vous avez consulté ?	◯ 1e<br>◯ 2e<br>◯ 3e<br>◯ 4e<br>◯ 5e
S'agit-il d'une nouvelle visite, mais d'un prestataire pour lequel vous avez déjà rempli un trajet de soins ?	◯ Non<br>◯ Oui, même que trajet de soins 1<br>◯ Oui, même que trajet de soins 2<br>◯ Oui, même que trajet de soins 3<br>◯ Oui, même que trajet de soins 4<br>◯ Oui, même que trajet de soins 5
Qui avez-vous consulté ou qui a donné les médicaments ?	◯ Tradipraticien (bokoko/nganga/féticheur/église)<br>◯ Pharmacie privée ou vendeur des médicaments<br>◯ Relais communautaire<br>◯ Poste de santé<br>◯ Centre de santé privé<br>◯ Centre de santé des structures de référence<br>◯ Prestataire privé<br>◯ Je ne sais pas
Est-ce que c'est ce prestataire qui vous a référé à l'hôpital ?	◯ Oui, pendant cette visite<br>◯ Oui, mais pendant une visite suivante<br>◯ Non
Quand avez-vous fait ça ?<br>Cliquez sur le calendrier et sélectionnez la date correcte	______________________________
Si la date est inconnu	◯ Je ne sais pas
Est-ce que c'était le matin, l'après-midi ou le soir/la nuit ?	◯ Matin (= après le lever de soleil)<br>◯ Après-midi (= après 12 h)<br>◯ Soir ou nuit jusqu'à minuit (= après le coucher de soleil)<br>◯ Nuit après minuit (= avant le lever de soleil)<br>◯ Je ne sais pas
Avez-vous passer la nuit là-bas ?	◯ Oui<br>◯ Non
Si oui, jusqu'à quand ?	______________________________
Jusqu'à le matin, l'après-midi ou le soir/la nuit ?	◯ Matin (= après le lever de soleil)<br>◯ Après-midi (= après 12 h)<br>◯ Soir ou nuit jusqu'à minuit (= après le coucher de soleil)<br>◯ Nuit après minuit (= avant le lever de soleil)<br>◯ Je ne sais pas

Est-ce que vous avez dû vous déplacer en dehors de votre village ou aire de santé ?	◯ Oui, mais nous sommes restés dans notre village<br>◯ Oui, autre village du même aire de santé<br>◯ Oui, autre aire de santé<br>◯ Non (resté à domicile)<br>◯ Je ne sais pas
Si vous avez dû vous déplacer, comment vous êtes allées là-bas ?	◯ A pied<br>◯ Vélo<br>◯ Moto personelle<br>◯ Mototaxi<br>◯ Voiture personelle<br>◯ Taxi<br>◯ Ambulance<br>◯ Je ne sais pas
Si autre, préciser :	______________________________
Pourquoi vous avez choisi cette manière d'y aller ou choisi de rester à domicile ?	☐ A cause de la distance<br>☐ De l'état de la route<br>☐ De la fiabilité<br>☐ Des coûts<br>☐ A cause de l'état de l'enfant<br>☐ A cause du nombre de personne<br>☐ Absence d'autre mode de transport<br>☐ Autre<br>☐ Je ne sais pas<br>(Cochez toutes les réponses applicables)
Autre, préciser :	______________________________
Est-ce qu'ils ont prélevé du sang de l'enfant pour faire des tests laboratoires ?	◯ Oui<br>◯ Non<br>◯ Je ne sais pas
Est-ce qu'ils ont donné un traitement à l'enfant ?	◯ Oui<br>◯ Non<br>◯ Je ne sais pas
Combien de traitements ?	______________________________
Nombre de traitements inconnu :	◯ Je ne sais pas
Combien de traitements connu (classe et nom de produit, voie d'administration) ?<br>10 max.	______________________________

Produit #1

Classe de produit	◯ Traitement traditionnel<br>◯ Antipaludéen<br>◯ Antibiotique<br>◯ Antipyrétique<br>◯ Solution de réhydratation orale<br>◯ Sérum (glucosé/fysiologique/Ringer Lactate/...)<br>◯ Transfusion sanguine<br>◯ Fer<br>◯ Vitamines/acide folique<br>◯ Combinaison de fer et vitamines/acide folique<br>◯ Vermicide<br>◯ Autre<br>◯ Inconnu
Nom du produit connu ?	◯ Oui<br>◯ Non
Nom du produit	__________________________________
Voie d'administration	◯ Application locale<br>◯ Voie orale<br>◯ Intrarectal<br>◯ Injection intramusculaire<br>◯ Injection intraveineuse<br>◯ Application locale + orale<br>◯ Autre<br>◯ Inconnu
Autre, préciser	__________________________________
Traitement déjà arreté ?	◯ Oui<br>◯ Non<br>◯ Je ne sais pas
Date d'arrêt	__________________________________
Temps d'arrêt	◯ Matin (= après le lever de soleil)<br>◯ Après-midi (= après 12 h)<br>◯ Soir ou nuit jusqu'à minuit (= après le coucher de soleil)<br>◯ Nuit après minuit (= avant le lever de soleil)<br>◯ Je ne sais pas

Produit #2

Classe de produit	◯ Traitement traditionnel<br>◯ Antipaludéen<br>◯ Antibiotique<br>◯ Antipyrétique<br>◯ Solution de réhydratation orale<br>◯ Sérum (glucosé/fysiologique/Ringer Lactate/...)<br>◯ Transfusion sanguine<br>◯ Fer<br>◯ Vitamines/acide folique<br>◯ Combinaison de fer et vitamines/acide folique<br>◯ Vermicide<br>◯ Autre<br>◯ Inconnu
Nom du produit connu ?	◯ Oui<br>◯ Non
Nom du produit	__________________________
Voie d'administration	◯ Application locale<br>◯ Voie orale<br>◯ Intrarectal<br>◯ Injection intramusculaire<br>◯ Injection intraveineuse<br>◯ Application locale + orale<br>◯ Autre<br>◯ Inconnu
Autre, préciser	__________________________
Traitement déjà arreté ?	◯ Oui<br>◯ Non<br>◯ Je ne sais pas
Date d'arrêt	__________________________
Temps d'arrêt	◯ Matin (= après le lever de soleil)<br>◯ Après-midi (= après 12 h)<br>◯ Soir ou nuit jusqu'à minuit (= après le coucher de soleil)<br>◯ Nuit après minuit (= avant le lever de soleil)<br>◯ Je ne sais pas

Produit #3

Classe de produit	◯ Traitement traditionnel<br>◯ Antipaludéen<br>◯ Antibiotique<br>◯ Antipyrétique<br>◯ Solution de réhydratation orale<br>◯ Sérum (glucosé/fysiologique/Ringer Lactate/...)<br>◯ Transfusion sanguine<br>◯ Fer<br>◯ Vitamines/acide folique<br>◯ Combinaison de fer et vitamines/acide folique<br>◯ Vermicide<br>◯ Autre<br>◯ Inconnu
Nom du produit connu ?	◯ Oui<br>◯ Non
Nom du produit	__________________________________
Voie d'administration	◯ Application locale<br>◯ Voie orale<br>◯ Intrarectal<br>◯ Injection intramusculaire<br>◯ Injection intraveineuse<br>◯ Application locale + orale<br>◯ Autre<br>◯ Inconnu
Autre, préciser	__________________________________
Traitement déjà arreté ?	◯ Oui<br>◯ Non<br>◯ Je ne sais pas
Date d'arrêt	__________________________________
Temps d'arrêt	◯ Matin (= après le lever de soleil)<br>◯ Après-midi (= après 12 h)<br>◯ Soir ou nuit jusqu'à minuit (= après le coucher de soleil)<br>◯ Nuit après minuit (= avant le lever de soleil)<br>◯ Je ne sais pas

Produit #4

Classe de produit	◯ Traitement traditionnel<br>◯ Antipaludéen<br>◯ Antibiotique<br>◯ Antipyrétique<br>◯ Solution de réhydratation orale<br>◯ Sérum (glucosé/fysiologique/Ringer Lactate/...)<br>◯ Transfusion sanguine<br>◯ Fer<br>◯ Vitamines/acide folique<br>◯ Combinaison de fer et vitamines/acide folique<br>◯ Vermicide<br>◯ Autre<br>◯ Inconnu
Nom du produit connu ?	◯ Oui<br>◯ Non
Nom du produit	__________________________________
Voie d'administration	◯ Application locale<br>◯ Voie orale<br>◯ Intrarectal<br>◯ Injection intramusculaire<br>◯ Injection intraveineuse<br>◯ Application locale + orale<br>◯ Autre<br>◯ Inconnu
Autre, préciser	__________________________________
Traitement déjà arreté ?	◯ Oui<br>◯ Non<br>◯ Je ne sais pas
Date d'arrêt	__________________________________
Temps d'arrêt	◯ Matin (= après le lever de soleil)<br>◯ Après-midi (= après 12 h)<br>◯ Soir ou nuit jusqu'à minuit (= après le coucher de soleil)<br>◯ Nuit après minuit (= avant le lever de soleil)<br>◯ Je ne sais pas

Produit #5

Classe de produit	◯ Traitement traditionnel<br>◯ Antipaludéen<br>◯ Antibiotique<br>◯ Antipyrétique<br>◯ Solution de réhydratation orale<br>◯ Sérum (glucosé/fysiologique/Ringer Lactate/...)<br>◯ Transfusion sanguine<br>◯ Fer<br>◯ Vitamines/acide folique<br>◯ Combinaison de fer et vitamines/acide folique<br>◯ Vermicide<br>◯ Autre<br>◯ Inconnu
Nom du produit connu ?	◯ Oui<br>◯ Non
Nom du produit	__________________________
Voie d'administration	◯ Application locale<br>◯ Voie orale<br>◯ Intrarectal<br>◯ Injection intramusculaire<br>◯ Injection intraveineuse<br>◯ Application locale + orale<br>◯ Autre<br>◯ Inconnu
Autre, préciser	__________________________
Traitement déjà arreté ?	◯ Oui<br>◯ Non<br>◯ Je ne sais pas
Date d'arrêt	__________________________
Temps d'arrêt	◯ Matin (= après le lever de soleil)<br>◯ Après-midi (= après 12 h)<br>◯ Soir ou nuit jusqu'à minuit (= après le coucher de soleil)<br>◯ Nuit après minuit (= avant le lever de soleil)<br>◯ Je ne sais pas

Produit #6

Classe de produit	◯ Traitement traditionnel<br>◯ Antipaludéen<br>◯ Antibiotique<br>◯ Antipyrétique<br>◯ Solution de réhydratation orale<br>◯ Sérum (glucosé/fysiologique/Ringer Lactate/...)<br>◯ Transfusion sanguine<br>◯ Fer<br>◯ Vitamines/acide folique<br>◯ Combinaison de fer et vitamines/acide folique<br>◯ Vermicide<br>◯ Autre<br>◯ Inconnu
Nom du produit connu ?	◯ Oui<br>◯ Non
Nom du produit	__________________________
Voie d'administration	◯ Application locale<br>◯ Voie orale<br>◯ Intrarectal<br>◯ Injection intramusculaire<br>◯ Injection intraveineuse<br>◯ Application locale + orale<br>◯ Autre<br>◯ Inconnu
Autre, préciser	__________________________
Traitement déjà arreté ?	◯ Oui<br>◯ Non<br>◯ Je ne sais pas
Date d'arrêt	__________________________
Temps d'arrêt	◯ Matin (= après le lever de soleil)<br>◯ Après-midi (= après 12 h)<br>◯ Soir ou nuit jusqu'à minuit (= après le coucher de soleil)<br>◯ Nuit après minuit (= avant le lever de soleil)<br>◯ Je ne sais pas

Produit #7

Classe de produit	◯ Traitement traditionnel<br>◯ Antipaludéen<br>◯ Antibiotique<br>◯ Antipyrétique<br>◯ Solution de réhydratation orale<br>◯ Sérum (glucosé/fysiologique/Ringer Lactate/...)<br>◯ Transfusion sanguine<br>◯ Fer<br>◯ Vitamines/acide folique<br>◯ Combinaison de fer et vitamines/acide folique<br>◯ Vermicide<br>◯ Autre<br>◯ Inconnu
Nom du produit connu ?	◯ Oui<br>◯ Non
Nom du produit	__________________________
Voie d'administration	◯ Application locale<br>◯ Voie orale<br>◯ Intrarectal<br>◯ Injection intramusculaire<br>◯ Injection intraveineuse<br>◯ Application locale + orale<br>◯ Autre<br>◯ Inconnu
Autre, préciser	__________________________
Traitement déjà arreté ?	◯ Oui<br>◯ Non<br>◯ Je ne sais pas
Date d'arrêt	__________________________
Temps d'arrêt	◯ Matin (= après le lever de soleil)<br>◯ Après-midi (= après 12 h)<br>◯ Soir ou nuit jusqu'à minuit (= après le coucher de soleil)<br>◯ Nuit après minuit (= avant le lever de soleil)<br>◯ Je ne sais pas

Produit #8

Classe de produit	◯ Traitement traditionnel<br>◯ Antipaludéen<br>◯ Antibiotique<br>◯ Antipyrétique<br>◯ Solution de réhydratation orale<br>◯ Sérum (glucosé/fysiologique/Ringer Lactate/...)<br>◯ Transfusion sanguine<br>◯ Fer<br>◯ Vitamines/acide folique<br>◯ Combinaison de fer et vitamines/acide folique<br>◯ Vermicide<br>◯ Autre<br>◯ Inconnu
Nom du produit connu ?	◯ Oui<br>◯ Non
Nom du produit	__________________________
Voie d'administration	◯ Application locale<br>◯ Voie orale<br>◯ Intrarectal<br>◯ Injection intramusculaire<br>◯ Injection intraveineuse<br>◯ Application locale + orale<br>◯ Autre<br>◯ Inconnu
Autre, préciser	__________________________
Traitement déjà arreté ?	◯ Oui<br>◯ Non<br>◯ Je ne sais pas
Date d'arrêt	__________________________
Temps d'arrêt	◯ Matin (= après le lever de soleil)<br>◯ Après-midi (= après 12 h)<br>◯ Soir ou nuit jusqu'à minuit (= après le coucher de soleil)<br>◯ Nuit après minuit (= avant le lever de soleil)<br>◯ Je ne sais pas

Produit #9

Classe de produit	◯ Traitement traditionnel<br>◯ Antipaludéen<br>◯ Antibiotique<br>◯ Antipyrétique<br>◯ Solution de réhydratation orale<br>◯ Sérum (glucosé/fysiologique/Ringer Lactate/...)<br>◯ Transfusion sanguine<br>◯ Fer<br>◯ Vitamines/acide folique<br>◯ Combinaison de fer et vitamines/acide folique<br>◯ Vermicide<br>◯ Autre<br>◯ Inconnu
Nom du produit connu ?	◯ Oui<br>◯ Non
Nom du produit	______________________________
Voie d'administration	◯ Application locale<br>◯ Voie orale<br>◯ Intrarectal<br>◯ Injection intramusculaire<br>◯ Injection intraveineuse<br>◯ Application locale + orale<br>◯ Autre<br>◯ Inconnu
Autre, préciser	______________________________
Traitement déjà arreté ?	◯ Oui<br>◯ Non<br>◯ Je ne sais pas
Date d'arrêt	______________________________
Temps d'arrêt	◯ Matin (= après le lever de soleil)<br>◯ Après-midi (= après 12 h)<br>◯ Soir ou nuit jusqu'à minuit (= après le coucher de soleil)<br>◯ Nuit après minuit (= avant le lever de soleil)<br>◯ Je ne sais pas

Produit #10

Classe de produit	◯ Traitement traditionnel<br>◯ Antipaludéen<br>◯ Antibiotique<br>◯ Antipyrétique<br>◯ Solution de réhydratation orale<br>◯ Sérum (glucosé/fysiologique/Ringer Lactate/...)<br>◯ Transfusion sanguine<br>◯ Fer<br>◯ Vitamines/acide folique<br>◯ Combinaison de fer et vitamines/acide folique<br>◯ Vermicide<br>◯ Autre<br>◯ Inconnu
Nom du produit connu ?	◯ Oui<br>◯ Non
Nom du produit	______________________
Voie d'administration	◯ Application locale<br>◯ Voie orale<br>◯ Intrarectal<br>◯ Injection intramusculaire<br>◯ Injection intraveineuse<br>◯ Application locale + orale<br>◯ Autre<br>◯ Inconnu
Autre, préciser	______________________
Traitement déjà arreté ?	◯ Oui<br>◯ Non<br>◯ Je ne sais pas
Date d'arrêt	______________________
Temps d'arrêt	◯ Matin (= après le lever de soleil)<br>◯ Après-midi (= après 12 h)<br>◯ Soir ou nuit jusqu'à minuit (= après le coucher de soleil)<br>◯ Nuit après minuit (= avant le lever de soleil)<br>◯ Je ne sais pas

HIT Lettre de reference

Sélectez votre nom	◯ Adèle Zomba Lutumba<br>◯ Emmanuel Ntangu Bamikina<br>◯ Grace Kasidikoko<br>◯ Irene Kimbembi Mansosa<br>◯ Japhet Ngina Mbala<br>◯ Naomi Wasolua<br>◯ Naomie Nama Mukenyi<br>◯ Nathalie Ndengila<br>◯ Thomas Nsema Mbaki

LETTRE DE RÉFÉRENCE

Référence motivée par un lettre de référence ?	◯ Oui<br>◯ Non
Date de référence	__________________________
Date de référence pas noté	◯ Pas noté
Heure de référence	__________________________
Heure de référence pas noté	◯ Pas noté
Date de l'arrivée à l'hôpital	__________________________
Date de l'arrivée pas noté	◯ Pas noté
Heure de l'arrivée à l'hôpital	__________________________
Heure de l'arrivée pas noté	◯ Pas noté

Centre de santé qui a référée	◯ Nkandu 1<br>◯ Nkandu 2<br>◯ Kasa Vubu<br>◯ Ndinga Mbote<br>◯ Nkandu 3<br>◯ Gare<br>◯ Snel/Inkisi<br>◯ Kikonka 1<br>◯ Kikonka 2<br>◯ Kitanu/Etat<br>◯ Cerphytoco<br>◯ Omeco<br>◯ Wete<br>◯ Ngeba<br>◯ Kavuaya<br>◯ St. Pierre Boko<br>◯ Kimuisi<br>◯ Kinkoko<br>◯ Kipako<br>◯ Kituengi<br>◯ Kipasa<br>◯ Kivuangi<br>◯ Lemfu<br>◯ Madimba/Etat<br>◯ Cederi<br>◯ Kilenda<br>◯ Yimbi<br>◯ Pides<br>◯ Pas noté<br>◯ Autre
Autre, préciser	__________________________________
Mutualiste	◯ Oui<br>◯ Non<br>◯ Pas noté
Plaintes principales (plusiers options possible)	☐ Fievre<br>☐ Anorexie<br>☐ Asthénie<br>☐ Anémie<br>☐ Vomissements<br>☐ Diarrée<br>☐ Douleur abdominal<br>☐ Toux<br>☐ Cephalées<br>☐ Convulsion<br>☐ Pleures incessants<br>☐ Paleur palmaire<br>☐ Détresse respiratoire / dyspnee<br>☐ Autres<br>☐ Pas noté<br>(Cochez toutes les réponses applicables)
Autre, préciser	__________________________________
Diagnostique présomptive connu ?	◯ Oui<br>◯ Non<br>◯ Pas noté

Diagnostique présomptive (plusiers options possible)	☐ Paludisme grave<br>☐ Paludisme simple<br>☐ Pneumonie<br>☐ Sepsis<br>☐ Méningite<br>☐ Anémie<br>☐ Déshydratation<br>☐ Malnutrition aigüe sévère<br>☐ Autre<br>☐ Pas noté<br>(Cochez toutes les réponses applicables)
Autre, préciser	__________________________________
Numéro porte d'entrée ordinogramme connu	◯ Oui<br>◯ Non<br>◯ Pas noté
Numéro porte d'entrée ordinogramme	__________________________________
Etat du malade au moment de la référence (plusiers options possible)	☐ Bon<br>☐ Altéré<br>☐ Agité<br>☐ Désespéré<br>☐ Comateux<br>☐ Autre<br>☐ Pas noté<br>(Cochez toutes les réponses applicables)
Autre, préciser	__________________________________
Motif de la référence (plusiers options possible)	☐ Urgence<br>☐ Sur demande du malade<br>☐ Précision diagnostique<br>☐ Pas de médicaments<br>☐ Echec thérapeutique<br>☐ Bonne prise en charge<br>☐ Autre<br>☐ Pas noté<br>(Cochez toutes les réponses applicables)
Autre, préciser	__________________________________

HIT Suivi quotidien

Le patient participe-t-il à l'étude DeNTS ?	◯ Oui<br>◯ Non

Si le patient est inclus dans l'étude DeNTS, saisissez les données de laboratoire dans le CRF DeNTS.

HIT Diagnostic rapide

Sélectez votre nom	◯ Adèle Zomba Lutumba<br>◯ Emmanuel Ntangu Bamikina<br>◯ Grace Kasidikoko<br>◯ Irene Kimbembi Mansosa<br>◯ Japhet Ngina Mbala<br>◯ Naomi Wasolua<br>◯ Naomie Nama Mukenyi<br>◯ Nathalie Ndengila<br>◯ Thomas Nsema Mbaki

TEST DIAGNOSTIQUE RAPIDE PALUDISME

Test diagnostique rapide paludisme	◯ Fait<br>◯ Non fait
Date des tests de diagnostic rapide	______________________
TDR paludisme : ligne de contrôle	◯ Positif<br>◯ Négatif<br>◯ Pas noté
TDR paludisme : ligne Pan	◯ Positif<br>◯ Négatif<br>◯ Pas noté
TDR paludisme : ligne Pf	◯ Positif<br>◯ Négatif<br>◯ Pas noté, mais TDR positif<br>◯ Pas noté, mais TDR negatif

HIT Resultats goutte epaisse

Sélectez votre nom	◯ Adèle Zomba Lutumba<br>◯ Emmanuel Ntangu Bamikina<br>◯ Grace Kasidikoko<br>◯ Irene Kimbembi Mansosa<br>◯ Japhet Ngina Mbala<br>◯ Naomi Wasolua<br>◯ Naomie Nama Mukenyi<br>◯ Nathalie Ndengila<br>◯ Thomas Nsema Mbaki

GOUTTE ÉPAISSE POUR DÉTECTER LE PALUDISME

Goutte épaisse pour détecter le paludisme	◯ Fait<br>◯ Non fait
Date du prélèvement goutte épaisse	______________________
Date de l'examen de la goutte épaisse	______________________
Résultat goutte épaisse	◯ Positif<br>◯ Négatif<br>◯ Pas noté
Nombre de globules blancs comptés	______________________
Nombre de parasites comptés	______________________
Si positif : parasitémie	______________________<br>(/µL)
Si positif : présence de gamétocytes	◯ Oui<br>◯ Non
Quelle espèce ?	☐ Plasmodium falciparum<br>☐ Plasmodium malariae<br>☐ Plasmodium ovale<br>☐ Plasmodium knowlesi<br>☐ Plasmodium vivax<br>☐ Pas noté

HIT Resultats hemoculture

Sélectez votre nom
- ◯ Adèle Zomba Lutumba
- ◯ Emmanuel Ntangu Bamikina
- ◯ Grace Kasidikoko
- ◯ Irene Kimbembi Mansosa
- ◯ Japhet Ngina Mbala
- ◯ Naomi Wasolua
- ◯ Naomie Nama Mukenyi
- ◯ Nathalie Ndengila
- ◯ Thomas Nsema Mbaki

HÉMOCULTURE

Hémoculture arrivée au labo ?
- ◯ Oui
- ◯ Non

Numéro d'hémoculture ________________________________

Date du prélèvement de l'hémoculture ________________________________

Virage
- ◯ Oui
- ◯ Non

Si oui : date de virage ________________________________

Si oui : date d'identification définitive ________________________________

Si oui : identification définitive
- ☐ Salmonella spp
- ☐ Salmonella Typhi
- ☐ Staphylococcus aureus
- ☐ Escherichia coli/paracoli
- ☐ Klebsiella spp
- ☐ Contaminant
- ☐ Virage sans croissance
- ☐ Autre

(Cochez toutes les réponses applicables)

Autre, préciser ________________________________

HIT Issue

Séléctez votre nom	◯ Adèle Zomba Lutumba<br>◯ Emmanuel Ntangu Bamikina<br>◯ Grace Kasidikoko<br>◯ Irene Kimbembi Mansosa<br>◯ Japhet Ngina Mbala<br>◯ Naomi Wasolua<br>◯ Naomie Nama Mukenyi<br>◯ Nathalie Ndengila<br>◯ Thomas Nsema Mbaki
Indiquez l'issue de l'enfant basé sur sa fiche clinique de routine	◯ Évasion<br>◯ Sortie de l'hôpital<br>◯ Décès<br>◯ Retiré de l'étude
Indiquez la date de retrait/décès/sortie/évasion/fin de participation de l'enfant :	__________________________________
Si sortie de l'hôpital : quelle catégorie de sortie ?	◯ Référence<br>◯ Sortie sur avis médicale<br>◯ Sortie sur demande d'accompagnant
Raison de retrait de l'étude :	__________________________________

Cds Presentation Clinique

Sélectez votre nom	◯ Adèle Zomba Lutumba<br>◯ Emmanuel Ntangu Bamikina<br>◯ Grace Kasidikoko<br>◯ Irene Kimbembi Mansosa<br>◯ Japhet Ngina Mbala<br>◯ Naomi Wasolua<br>◯ Naomie Nama Mukenyi<br>◯ Nathalie Ndengila<br>◯ Thomas Nsema Mbaki
Nombre de visites au centre de santé (y inclus la visite dans laquelle l'enfant a été référée)	______________________

DONNEES DES FICHES CLINIQUES DES CENTRES DE SANTE QUI ONT REFERE L'ENFANT

Les fichiers cliniques ont-ils été trouvés	◯ Oui, un dossier par visite<br>◯ Oui, mais différents visites combinées dans un seul dossier<br>◯ Non
Date de la visite	______________________
Heure de la visite	______________________<br>(Si l'heure est inconnue, entrez 01:01)
Présence des signes de danger ?	◯ Oui<br>◯ Non<br>◯ Pas noté
Si oui, lesquels (plusiers options possible)	☐ Incapacité de boire ou manger<br>☐ Vomit tout ce qu'il consomme<br>☐ Convulsions récentes<br>☐ Convulsions actuelles<br>☐ Léthargique ou inconscient<br>☐ Pas noté<br>(Cochez toutes les réponses applicables)
Maladie qui s'empire/ne s'améliore pas après une bonne prise en charge et qui alors nécessite la référence ?	◯ Oui<br>◯ Non<br>◯ Pas noté
Si oui, de quelle maladie s'agit-il ? (plusiers options possible)	☐ Anémie<br>☐ Difficultés respiratoires<br>☐ Déshydratation<br>☐ Diarrhée<br>☐ Fièvre<br>☐ Malnutrition<br>☐ Paludisme<br>☐ Autre<br>☐ Pas noté<br>(Cochez toutes les réponses applicables)
Autre, préciser	______________________

Difficultés respiratoires

Toux ou difficultés respiratoires
◯ Oui
◯ Non
◯ Pas noté

Fréquence respiratoire (respirations/minute)
____
(Si pas noté, entrez "-9")

Respiration rapide
◯ Oui
◯ Non
◯ Pas noté

Tirage sous-costale
◯ Oui
◯ Non
◯ Pas noté

Stridor
◯ Oui
◯ Non
◯ Pas noté

Sifflements
◯ Oui
◯ Non
◯ Pas noté

Diarrhée

Diarrhée
◯ Oui
◯ Non
◯ Pas noté

Sang dans les selles
◯ Oui
◯ Non
◯ Pas noté

Enfant agité ou irritable
◯ Oui
◯ Non
◯ Pas noté

Yeux enfoncés
◯ Oui
◯ Non
◯ Pas noté

Enfant assoiffé / qui boit avec avidité
◯ Oui
◯ Non
◯ Pas noté

Pli cutané qui s'efface après > 2 secondes
◯ Oui
◯ Non
◯ Pas noté

Fièvre

Fièvre	◯ Oui<br>◯ Non<br>◯ Pas noté
Si oui, fièvre depuis combien de jours	______________________<br>(Si pas noté, entrez "-9")
Si depuis 7 jours, fièvre présente chaque jour	◯ Oui<br>◯ Non<br>◯ Pas noté
Raideur de la nuque	◯ Oui<br>◯ Non<br>◯ Pas noté
Signes de rougeole : éruption cutanée généralisée accompagnée par le nez qui coule, la toux ou les yeux rouges	◯ Oui<br>◯ Non<br>◯ Pas noté
Rougeole pendant les 3 mois passées avec présence actuelle d'ulcérations orales, de pus qui coule des yeux ou d'opacité de la cornée	◯ Oui<br>◯ Non<br>◯ Pas noté

Problème d'oreille

Gonflement douloureux derrière l'oreille	◯ Oui<br>◯ Non<br>◯ Pas noté

Signes d'anémie

Pâleur palmaire	◯ Oui, sévère<br>◯ Oui, légère<br>◯ Oui, sévérité pas spécifié<br>◯ Non<br>◯ Pas noté
Drépanocytaire	◯ Oui<br>◯ Non<br>◯ Pas noté

Signes de malnutrition

Signes d'amaigrissement visible et sévère	◯ Oui<br>◯ Non<br>◯ Pas noté
Œdèmes nutritionnels	◯ Oui<br>◯ Non<br>◯ Pas noté
Si présence d'œdèmes, prenant le godet	◯ Oui<br>◯ Non<br>◯ Pas noté

Si présence d'œdèmes, œdèmes bilatérales	◯ Oui<br>◯ Non<br>◯ Pas noté
Rapport poids-taille (P/T)	◯ < -3 ET<br>◯ >= -3 et < -2 ET<br>◯ >= -2 ET<br>◯ Pas noté
Périmètre brachiale (PB)	◯ < 115 mm<br>◯ >= 115 - < 125 mm<br>◯ >= 125 mm<br>◯ Pas noté

Cds Processus Diagnostique

PALUDISME

Test diagnostique rapide paludisme
- ◯ Fait
- ◯ Non fait
- ◯ Pas noté

Résultat TDR paludisme
- ◯ Positif
- ◯ Négatif
- ◯ Invalide
- ◯ Pas noté

Goutte épaisse
- ◯ Fait
- ◯ Non fait
- ◯ Pas noté

Résultat goutte épaisse
- ◯ Positif
- ◯ Négatif
- ◯ Pas noté

HEMOGLOBINE

Hémoglobine
- ◯ Fait
- ◯ Non fait
- ◯ Pas noté

Résultat (g/dl)

____
(g/dl)

Méthode
- ◯ Hemocue 301
- ◯ Hemocue 801
- ◯ Hemocue 201
- ◯ Echelle colorimétrique OMS
- ◯ Methode Sahli
- ◯ Methode Lovibond
- ◯ Autre

Autre méthode, préciser

____

HEMATOCRIT

Hématocrit
- ◯ Fait
- ◯ Non fait
- ◯ Pas noté

Résultat (%)

____
(%)

Méthode
- ◯ Centrifugation
- ◯ Autre

Autre méthode, préciser

____

DIAGNOSTIQUE PRESOMPTIVE

Diagnostique présomptive (plusiers options possible)

- ☐ Paludisme sans spécification de la sévérité
- ☐ Paludisme grave
- ☐ Paludisme simple
- ☐ Pneumonie
- ☐ Sepsis
- ☐ Méningite
- ☐ Anémie
- ☐ Déshydratation
- ☐ Malnutrition aiguë sévère
- ☐ Gastroentérite (febrile)
- ☐ Fièvre typhoide / salmonellose
- ☐ Fièvre prolongée
- ☐ Syndrome infectieux
- ☐ Autre
- ☐ Pas noté

(Cochez toutes les réponses applicables)

Autre, préciser

____

Cds Traitement

Est-ce qu'ils ont donné un traitement à l'enfant ?	◯ Oui<br>◯ Non<br>◯ Pas noté
Si oui, combien de produits différent ont-ils donné ?	______________________
**Produit #1**	
Classe de produit	◯ Traitement traditionnel<br>◯ Antipaludéen<br>◯ Antibiotique<br>◯ Antipyrétique<br>◯ Solution de réhydratation orale<br>◯ Sérum (glucosé/fysiologique/Ringer Lactate/...)<br>◯ Transfusion sanguine<br>◯ Fer<br>◯ Vitamines/acide folique<br>◯ Combinaison de fer et vitamines/acide folique<br>◯ Vermicide<br>◯ Autre<br>◯ Inconnu
Nom du produit	______________________
Voie d'administration	◯ Application locale<br>◯ Voie orale<br>◯ Intrarectal<br>◯ Injection intramusculaire<br>◯ Injection intraveineuse<br>◯ Application locale + orale<br>◯ Autre<br>◯ Inconnu
Autre voie d'administration, préciser	______________________
Date de prescription	______________________
Nombre de jours de traitement préscrit	______________________<br>(Si pas noté, entrez "-9")

Produit #2

Classe de produit

- ◯ Traitement traditionnel
- ◯ Antipaludéen
- ◯ Antibiotique
- ◯ Antipyrétique
- ◯ Solution de réhydratation orale
- ◯ Sérum (glucosé/fysiologique/Ringer Lactate/...)
- ◯ Transfusion sanguine
- ◯ Fer
- ◯ Vitamines/acide folique
- ◯ Combinaison de fer et vitamines/acide folique
- ◯ Vermicide
- ◯ Autre
- ◯ Inconnu

Nom du produit

____

Voie d'administration

- ◯ Application locale
- ◯ Voie orale
- ◯ Intrarectal
- ◯ Injection intramusculaire
- ◯ Injection intraveineuse
- ◯ Application locale + orale
- ◯ Autre
- ◯ Inconnu

Autre voie d'administration, préciser

____

Date de prescription

____

Nombre de jours de traitement préscrit

____
(Si pas noté, entrez "-9")

Produit #3

Classe de produit

- ◯ Traitement traditionnel
- ◯ Antipaludéen
- ◯ Antibiotique
- ◯ Antipyrétique
- ◯ Solution de réhydratation orale
- ◯ Sérum (glucosé/fysiologique/Ringer Lactate/...)
- ◯ Transfusion sanguine
- ◯ Fer
- ◯ Vitamines/acide folique
- ◯ Combinaison de fer et vitamines/acide folique
- ◯ Vermicide
- ◯ Autre
- ◯ Inconnu

Nom du produit

____

Voie d'administration	◯ Application locale<br>◯ Voie orale<br>◯ Intrarectal<br>◯ Injection intramusculaire<br>◯ Injection intraveineuse<br>◯ Application locale + orale<br>◯ Autre<br>◯ Inconnu
Autre voie d'administration, préciser	__________________________________
Date de prescription	__________________________________
Nombre de jours de traitement préscrit	__________________________________<br>(Si pas noté, entrez "-9")

Produit #4

Classe de produit	◯ Traitement traditionnel<br>◯ Antipaludéen<br>◯ Antibiotique<br>◯ Antipyrétique<br>◯ Solution de réhydratation orale<br>◯ Sérum (glucosé/fysiologique/Ringer Lactate/...)<br>◯ Transfusion sanguine<br>◯ Fer<br>◯ Vitamines/acide folique<br>◯ Combinaison de fer et vitamines/acide folique<br>◯ Vermicide<br>◯ Autre<br>◯ Inconnu
Nom du produit	__________________________________
Voie d'administration	◯ Application locale<br>◯ Voie orale<br>◯ Intrarectal<br>◯ Injection intramusculaire<br>◯ Injection intraveineuse<br>◯ Application locale + orale<br>◯ Autre<br>◯ Inconnu
Autre voie d'administration, préciser	__________________________________
Date de prescription	__________________________________
Nombre de jours de traitement préscrit	__________________________________<br>(Si pas noté, entrez "-9")

Produit #5

Classe de produit

- ◯ Traitement traditionnel
- ◯ Antipaludéen
- ◯ Antibiotique
- ◯ Antipyrétique
- ◯ Solution de réhydratation orale
- ◯ Sérum (glucosé/fysiologique/Ringer Lactate/...)
- ◯ Transfusion sanguine
- ◯ Fer
- ◯ Vitamines/acide folique
- ◯ Combinaison de fer et vitamines/acide folique
- ◯ Vermicide
- ◯ Autre
- ◯ Inconnu

Nom du produit

____

Voie d'administration

- ◯ Application locale
- ◯ Voie orale
- ◯ Intrarectal
- ◯ Injection intramusculaire
- ◯ Injection intraveineuse
- ◯ Application locale + orale
- ◯ Autre
- ◯ Inconnu

Autre voie d'administration, préciser

____

Date de prescription

____

Nombre de jours de traitement préscrit

____
(Si pas noté, entrez "-9")

Produit #6

Classe de produit

- ◯ Traitement traditionnel
- ◯ Antipaludéen
- ◯ Antibiotique
- ◯ Antipyrétique
- ◯ Solution de réhydratation orale
- ◯ Sérum (glucosé/fysiologique/Ringer Lactate/...)
- ◯ Transfusion sanguine
- ◯ Fer
- ◯ Vitamines/acide folique
- ◯ Combinaison de fer et vitamines/acide folique
- ◯ Vermicide
- ◯ Autre
- ◯ Inconnu

Nom du produit

____

Voie d'administration

- ◯ Application locale
- ◯ Voie orale
- ◯ Intrarectal
- ◯ Injection intramusculaire
- ◯ Injection intraveineuse
- ◯ Application locale + orale
- ◯ Autre
- ◯ Inconnu

Autre voie d'administration, préciser

____

Date de prescription

____

Nombre de jours de traitement préscrit

____
(Si pas noté, entrez "-9")

Produit #7

Classe de produit

- ◯ Traitement traditionnel
- ◯ Antipaludéen
- ◯ Antibiotique
- ◯ Antipyrétique
- ◯ Solution de réhydratation orale
- ◯ Sérum (glucosé/fysiologique/Ringer Lactate/...)
- ◯ Transfusion sanguine
- ◯ Fer
- ◯ Vitamines/acide folique
- ◯ Combinaison de fer et vitamines/acide folique
- ◯ Vermicide
- ◯ Autre
- ◯ Inconnu

Nom du produit

____

Voie d'administration

- ◯ Application locale
- ◯ Voie orale
- ◯ Intrarectal
- ◯ Injection intramusculaire
- ◯ Injection intraveineuse
- ◯ Application locale + orale
- ◯ Autre
- ◯ Inconnu

Autre voie d'administration, préciser

____

Date de prescription

____

Nombre de jours de traitement préscrit

____
(Si pas noté, entrez "-9")

Produit #8

Classe de produit

- ◯ Traitement traditionnel
- ◯ Antipaludéen
- ◯ Antibiotique
- ◯ Antipyrétique
- ◯ Solution de réhydratation orale
- ◯ Sérum (glucosé/fysiologique/Ringer Lactate/...)
- ◯ Transfusion sanguine
- ◯ Fer
- ◯ Vitamines/acide folique
- ◯ Combinaison de fer et vitamines/acide folique
- ◯ Vermicide
- ◯ Autre
- ◯ Inconnu

Nom du produit

____

Voie d'administration

- ◯ Application locale
- ◯ Voie orale
- ◯ Intrarectal
- ◯ Injection intramusculaire
- ◯ Injection intraveineuse
- ◯ Application locale + orale
- ◯ Autre
- ◯ Inconnu

Autre voie d'administration, préciser

____

Date de prescription

____

Nombre de jours de traitement préscrit

____
(Si pas noté, entrez "-9")

Produit #9

Classe de produit

- ◯ Traitement traditionnel
- ◯ Antipaludéen
- ◯ Antibiotique
- ◯ Antipyrétique
- ◯ Solution de réhydratation orale
- ◯ Sérum (glucosé/fysiologique/Ringer Lactate/...)
- ◯ Transfusion sanguine
- ◯ Fer
- ◯ Vitamines/acide folique
- ◯ Combinaison de fer et vitamines/acide folique
- ◯ Vermicide
- ◯ Autre
- ◯ Inconnu

Nom du produit

____

Voie d'administration

- ◯ Application locale
- ◯ Voie orale
- ◯ Intrarectal
- ◯ Injection intramusculaire
- ◯ Injection intraveineuse
- ◯ Application locale + orale
- ◯ Autre
- ◯ Inconnu

Autre voie d'administration, préciser

____

Date de prescription

____

Nombre de jours de traitement préscrit

____
(Si pas noté, entrez "-9")

Produit #10

Classe de produit

- ◯ Traitement traditionnel
- ◯ Antipaludéen
- ◯ Antibiotique
- ◯ Antipyrétique
- ◯ Solution de réhydratation orale
- ◯ Sérum (glucosé/fysiologique/Ringer Lactate/...)
- ◯ Transfusion sanguine
- ◯ Fer
- ◯ Vitamines/acide folique
- ◯ Combinaison de fer et vitamines/acide folique
- ◯ Vermicide
- ◯ Autre
- ◯ Inconnu

Nom du produit

____

Voie d'administration

- ◯ Application locale
- ◯ Voie orale
- ◯ Intrarectal
- ◯ Injection intramusculaire
- ◯ Injection intraveineuse
- ◯ Application locale + orale
- ◯ Autre
- ◯ Inconnu

Autre voie d'administration, préciser

____

Date de prescription

____

Nombre de jours de traitement préscrit

____
(Si pas noté, entrez "-9")

Cds Reference

Est-ce qu'ils ont planifié un rendez-vous de suivi pour l'enfant ?	◯ Oui, date connu<br>◯ Oui, mais date inconnu<br>◯ Non<br>◯ Pas noté
Date du rendez-vous planifié	__________________________
Est-ce qu'ils ont décidé de référer l'enfant ?	◯ Oui<br>◯ Non<br>◯ Pas noté
Date de référence	__________________________
Heure de référence	__________________________
Pour quel motif ? (plusiers options possible)	☐ Urgence<br>☐ Sur demande du malade<br>☐ Précision diagnostique<br>☐ Pas de médicaments<br>☐ Echec thérapeutique<br>☐ Bonne prise en charge<br>☐ Autre<br>☐ Pas noté<br>(Cochez toutes les réponses applicables)
Autre motif :	__________________________
Date et heure de sortie du centre de santé	☐ Date connu<br>☐ Heure connu<br>☐ Inconnu<br>(Cochez tout ce qui s'applique)
Date de sortie du centre de santé	__________________________
Heure de sortie du centre de santé	__________________________
