# Supplementary material for: Health itinerary-related survival of children under-five with severe malaria or bloodstream infection, DR Congo
Source: PLoS Negl Trop Dis. 2023 Mar 6;17(3):e0011156. doi: 10.1371/journal.pntd.0011156 (PMC10019685; doi:10.1371/journal.pntd.0011156)
Supplement: S1 Fig — Twenty-one children were lost to follow-up due to hospital evasion or in-hospital referral: they were enrolled in the Kaplan-Meier curve but excluded from analysis in the table. (DOCX) [file pntd.0011156.s007.docx]

Supplementary Materials: Health itinerary-related survival of children under-five with severe malaria or bloodstream infection, DR Congo

Bieke Tack ^1,2,3^, Daniel Vita ^4^, José Nketo ^5^, Naomie Wasolua ^4^, Nathalie Ndengila ^4^, Natacha Herssens ^1^, Emmanuel Ntangu ^4^, Grace Kasidiko ^4^, Gaëlle Nkoji-Tunda ^6,7^, Marie-France Phoba ^6,7^, Justin Im ^8^, Hyon Jin Jeon ^8,9^, Florian Marks ^8-12^, Jaan Toelen ^3,9^, Octavie Lunguya ^6,7^ and Jan Jacobs ^1,2^

**Affiliations:**

1. Department of Clinical Sciences, Institute of Tropical Medicine, Antwerp, Belgium
2. Department of Microbiology, Immunology and Transplantation, KU Leuven, Leuven, Belgium
3. Department of Pediatrics, University Hospitals UZ Leuven, Leuven, Belgium
4. Hôpital Général de Référence Saint Luc de Kisantu, Kisantu, Democratic Republic of the Congo
5. Zone de Santé Kisantu, Kisantu, Democratic Republic of the Congo
6. Department of Microbiology, Institut National de Recherche Biomédicale, Kinshasa, Democratic Republic of the Congo
7. Department of Medical Biology, University Teaching Hospital of Kinshasa, Kinshasa, Democratic Republic of the Congo
8. International Vaccine Institute, Seoul, Republic of Korea
9. Cambridge Institute of Therapeutic Immunology and Infectious Disease, University of Cambridge School of Clinical Medicine, Cambridge, UK
10. Heidelberg Institute of Global Health, University of Heidelberg, Heidelberg, Germany
11. Madagascar Institute for Vaccine Research, University of Antananarivo, Antananarivo, Madagascar
12. Department of Development and Regeneration, KU Leuven, 3000 Leuven, Belgium

**Corresponding author:**

Bieke Tack, [btack@itg.be](mailto:btack@itg.be)

**Supplementary Fig S1.** In-hospital survival of children under-five admitted with severe febrile illness according to the duration of their health itinerary. Twenty-one children were lost to follow-up due to hospital evasion or in-hospital referral: they were enrolled in the Kaplan-Meier curve but excluded from analysis in the table. **
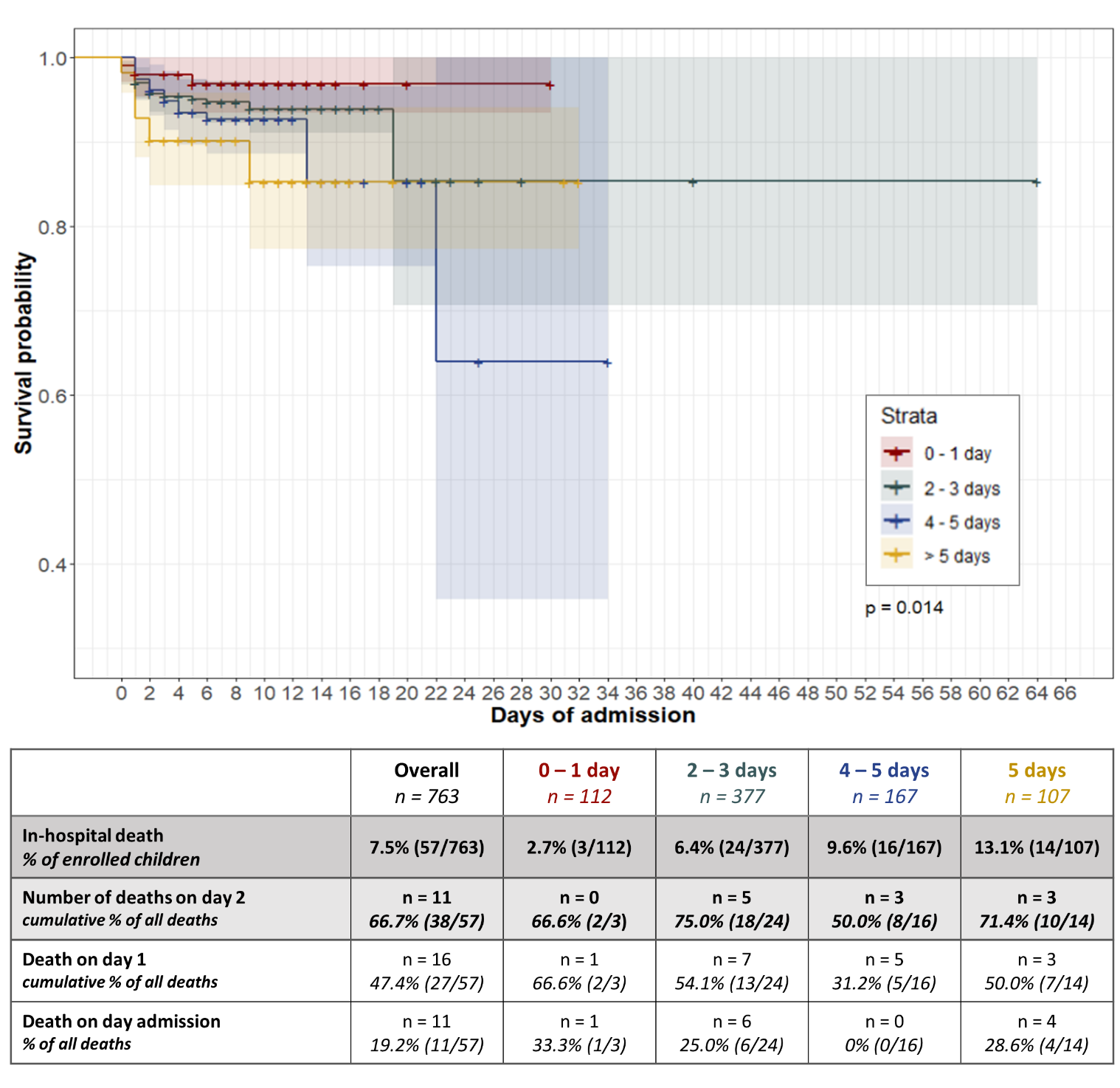
**
